# Multimodal network dynamics underpinning working memory

**DOI:** 10.1038/s41467-020-15541-0

**Published:** 2020-06-15

**Authors:** Andrew C. Murphy, Maxwell A. Bertolero, Lia Papadopoulos, David M. Lydon-Staley, Danielle S. Bassett

**Affiliations:** 1 Department of Bioengineering, School of Engineering & Applied Sciences, University of Pennsylvania, Philadelphia, PA 19104 USA; 2 Perelman School of Medicine, University of Pennsylvania, Philadelphia, PA 19104 USA; 3 Department of Physics and Astronomy, School of Arts & Sciences, University of Pennsylvania, Philadelphia, PA 19104 USA; 4 Department of Neurology, Perelman School of Medicine, University of Pennsylvania, Philadelphia, PA 19104 USA; 5 Department of Psychiatry, Perelman School of Medicine, University of Pennsylvania, Philadelphia, PA 19104 USA; 6 Department of Electrical & Systems Engineering, School of Engineering & Applied Sciences, University of Pennsylvania, Philadelphia, PA 19104 USA; 7 Santa Fe Institute, Santa Fe, NM 87501 USA

**Keywords:** Cognitive neuroscience, Computational neuroscience, Working memory

## Abstract

Complex human cognition arises from the integrated processing of multiple brain systems. However, little is known about how brain systems and their interactions might relate to, or perhaps even explain, human cognitive capacities. Here, we address this gap in knowledge by proposing a mechanistic framework linking frontoparietal system activity, default mode system activity, and the interactions between them, with individual differences in working memory capacity. We show that working memory performance depends on the strength of functional interactions between the frontoparietal and default mode systems. We find that this strength is modulated by the activation of two newly described brain regions, and demonstrate that the functional role of these systems is underpinned by structural white matter. Broadly, our study presents a holistic account of how regional activity, functional connections, and structural linkages together support integrative processing across brain systems in order for the brain to execute a complex cognitive process.

## Abstract (web summary)

Working memory is a critical component of executive function that allows people to complete complex tasks in the moment. Here, the authors show that this ability is underpinned by two newly defined brain networks.

## Introduction

While many cognitive processes can be mapped to individual brain regions, higher-order cognitive capacities necessarily depend on complex interactions across large-scale brain systems via regional activity, functional connections, and structural linkages. One example of a cognitive process that depends on multiple systems is working memory, which supports the short-term storage of information, thereby facilitating its further manipulation and processing^1^. Individual differences in working memory performance have been associated with differences in the recruitment of distinct brain systems, and in the functional interactions between such systems^2–4^. Working memory function is in part supported by the frontoparietal cortex, involved in cognitive control^5,6^, as well as the default-mode system, notable for its activation in the human resting state. Interestingly, these two systems are thought to have opposite effects on working memory: frontoparietal activity is vital for directing attention to external stimuli^7^, while default-mode activity is important for internally directed cognition^8^. Notably, the interactions between these two systems vary with cognitive state^9^, and play a critical role in goal-directed cognition^10^. General large-scale mechanisms of how brain systems interact to execute complex and integrative cognitive processing have been proposed^4,11,12^, where nodes in the frontoparietal network (i.e., connector nodes), via diverse connections across the brain systems and strong connections to each other, tune the connectivity between the brain’s distinct systems to achieve integrated cognition. However, it is unknown exactly how this occurs for a specific cognitive process between the brain systems known to subserve that process. Here, we address this gap in exactly how the frontoparietal network tunes brain connectivity by analyzing how the frontoparietal system interacts with and modulates the default-mode system during working memory.

Previous analyses have suggested that these two systems tend to be in functional competition during tasks with high working memory load; in such tasks, the activity of the two systems is anticorrelated^13^. This anticorrelation might enable maximally disjunctive levels of activity^13^. More simply, intersystem competition might allow for a pattern of whole-brain dynamics characterized by heightened activity in the frontoparietal system coupled with decreased activity in the default-mode system. Explaining such a pattern of dynamics is particularly important in light of evidence that it favors improved working memory performance^14^. However, while of course conceptually interesting, competition is not in itself a mechanism. Thus, here, we seek to address how competition may come about and the dynamic processes underlying competition. By illuminating possible causal pathways underpinning competition, a mechanistic framework would allow for the investigation and validation of cognitive arguments. Further, a mechanistic framing of competition may not only depend on functional brain network observations, but may also draw on the white matter structures subserving those functional dynamics. Structurally, it is well known that the two systems are quite distinct in terms of their topological role within the connectome: the default-mode system is part of the so-called rich club of the structural connectome, which is a set of densely interconnected high-degree nodes, while the frontoparietal system is part of the so-called diverse club, which is a set of densely interconnected nodes with diverse connectivity across all putative cognitive systems^11^. It is intuitively plausible that such distinct placements within the larger whole-brain network could constrain or define the roles that each system can play in inducing certain types of dynamics in general^15,16^, and competition in particular.

Here, we seek to perform a study of multimodal neural phenotypes, including regional activity, interregional connectivity, and structural linkages and their relationship to individual differences in working memory function. In particular, we seek to form a mechanistic framework to explain how these multimodal neural phenotypes produce competition between the frontoparietal and the default-mode system. We also seek to complement data-driven analysis of empirical measurements with biologically motivated computational modeling to probe the validity of our explanations and posited mechanisms. Specifically, in this study, we use functional magnetic resonance imaging data collected from 644 healthy adult human participants in the Human Connectome Project during the performance of a 2-back working memory task. To address potential structural drivers of our findings, we also use diffusion tensor imaging data acquired in the same participants. We begin by using a model-based scheme to uncover functional groups or subnetworks within the entire frontoparietal system, which we subsequently find to display distinguishable patterns of gene coexpression. Specifically, we find that the frontoparietal system can be fractioned into two nonoverlapping subnetworks, where one subnetwork is functionally aligned with the default-mode system, and the other subnetwork is functionally aligned with the dorsal attention system. We show that the relative activity level of these two subnetworks can push the frontoparietal system into functional alignment with either the default-mode or the dorsal attention system. We then demonstrate that the strength of structural linkages between the subnetworks and the dorsal attention and default-mode system mirrors the strength of the functional linkages. Last—drawing on methods from the physics of complex systems and the theory of nonlinear dynamical systems—we build a computational model of the system and its dynamics as a collection of coupled oscillators, with parameters and coupling architecture informed by the biological evidence we uncover. We use the model to probe which model parameters are able to produce the observed phenomena, and to test our hypotheses about the relationships between activity, anatomical connectivity, and functional interactions.

## Results

### Frontoparietal subnetworks modulate functional connectivity

**Fig. 1:**
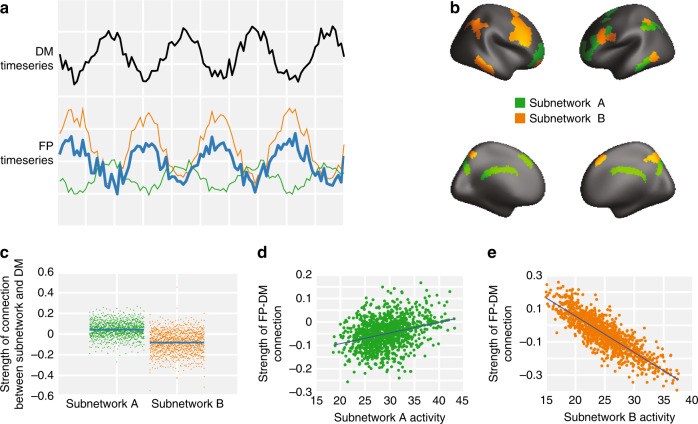
Subnetworks of the frontoparietal system. **a** We hypothesized that the strength of the connection between the frontoparietal and default-mode systems can be tuned by altering the relative amplitudes of subnetworks within the frontoparietal system. **b** Community detection reveals two distinct frontoparietal subnetworks, which we show here projected onto the cortical surface. **c** We found that the activity of subnetwork (A) was positively correlated with the activity of the default-mode system (mean *r* = 0.042, *p* < 0.001, *t*(1206) = 20, 95% CI: [0.038, 0.046]), while the activity of subnetwork (B) was negatively correlated with the activity of the default-mode system (mean *r* = −0.082, *p* < 0.001, *t*(1206) = −27, 95% CI: [−0.088, −0.076]). **d** Using a simple regression model, we tested whether the strength of the functional connection between the default-mode and frontoparietal systems could be predicted by a linear combination of the activity of subnetwork (A) and the activity of subnetwork (B). Within this model, we found that an increase in subnetwork (A) activity corresponds to an increase in the strength of the functional connection between the frontoparietal and default-mode systems (estimate of regression coefficient *β* = 0.006535, 95% CI: (0.00519, 0.00788), *p* < 0.001, *t*(1204) = 9.54). **e** Conversely, within the same model, we found that an increase in subnetwork (B) activity corresponds to a decrease in the strength of the functional connection between the two systems (estimate of regression coefficient *β* = −0.0112, 95% CI: (−0.01273, −0.0097), *p* < 0.001, *t*(1204) = −14.3).

We propose that the dynamics of the frontoparietal system directly modulate the strength of the functional connection between the frontoparietal and default-mode systems. For a discussion of contextualizing results and motivation for this specific choice, see Supplementary Notes 1 and 2. Our candidate mechanism assumes that the frontoparietal system is composed of two distinct, nonoverlapping subsystems. One network is posited to display dynamics that are correlated with the dynamics of the default-mode system, while the other subnetwork is posited to display dynamics that are anticorrelated with the dynamics of the default-mode system (Fig. 1a). The basis of this hypothesis was twofold: (1) there is prior work suggesting that the frontoparietal network is divisible into two subnetworks^17^, and (2) one possible parsimonious way for the subnetworks to arbitrarily tune the frontoparietal–default-mode system connection is for one subnetwork to be correlated with the default mode and the other to be anticorrelated with the default mode. Further, we propose that the relative activity magnitudes of these two systems interact to tune the strength of the intersystem (frontoparietal–default-mode) connection. When the first subnetwork is highly active relative to the second subnetwork, the strength of the intersystem connection will be positive; conversely, when the second subnetwork is highly active relative to the first subnetwork, the strength of the intersystem connection will be negative. Such an arrangement would allow for flexible tuning of the functional connection strength between the frontoparietal and default-mode systems. Importantly, this process would offer one possible mechanism to account for observed individual differences in frontoparietal–default-mode functional connection strength. Next, we hypothesize that two such subnetworks exist within the frontoparietal system, and we seek to identify them.

To assess the validity of this conceptual model, we first test the assumption that the frontoparietal system is composed of two distinct, spatially nonoverlapping subsystems. For each subject, we applied a weighted stochastic block model with *K* = 2 to the subgraph of the functional connectivity matrix representing functional connections between frontoparietal regions, and then we extracted a group-representative partition using a consensus similarity method^18^ (see “Methods”). The two subnetworks are shown on the cortex in Fig. 1b. To assess the statistical significance of this partition, we performed a nonparametric test, permuting the association of regions to subnetworks (see “Methods”). Against the null model, we found that this final consensus partition had a significantly higher log-likelihood using a multilevel model (*β* = −492.57, *p* < 0.0001, *t*(1769) = −71.0, *SE* = 6.9371, *n* = 2414). In addition, we fit the stochastic block model to our data using *K* = 1 and *K* = 3 to calculate the log-likelihood of the model fitting the data. We bootstrapped over subjects 10,000 times to calculate the difference in log-likelihood using *K* = 2 versus *K* = 1 or *K* = 3. Importantly, we found that the log-likelihood of the stochastic block model fits was significantly greater for *K* = 2 relative to *K* = 1 (*p* < 0.0001) and relative to *K* = 3 (*p* < 0.0001).

To further validate their biological distinctness, we sought to determine whether the two subnetworks showed distinguishable patterns of gene expression. To this end, we quantified the average magnitude of gene coexpression for pairs of regions for which both regions were located within a single subnetwork. We also quantified the average magnitude of gene coexpression for pairs of regions for which one region of the pair was located in one subnetwork, and the other region of the pair was located in the other subnetwork (see “Methods”). When tested against a nonparametric null model, we found that gene coexpression within subnetwork (A) was significantly higher than gene coexpression between subnetwork (A) and subnetwork (B) (*p* = 0.0057). Similarly, we found that gene coexpression within subnetwork (B) was higher than gene coexpression between subnetwork (B) and subnetwork (A) (*p* = 0.0111). For a complementary analyses demonstrating that this result cannot be explained by distances between regions, see Supplementary Note 6. For a complementary analysis discussing at which genes these subnetworks differ, see Supplementary Note 7. These findings support the notion that subnetwork (A) and subnetwork (B) are biologically distinct sectors of the frontoparietal system.

Next, we sought to test our proposition that—of the two frontoparietal subnetworks—one displays dynamics that are correlated with the dynamics of the default-mode system, while the other displays dynamics that are anticorrelated with those of the default-mode system. For each subject and each subnetwork, we calculated the strength of the functional connection between all pairs of regions for which one region of the pair is located in the subnetwork and the other region of the pair is located in the default-mode system. Consistent with our hypothesis, we found that subnetwork (A) is positively connected with the default-mode system (mean *r* = 0.042, *p* < 0.001 after Bonferroni correction for multiple comparisons, *t*(1206) = 20, 95% CI: [0.038, 0.046]; Fig. 1c left), while subnetwork (B) is negatively connected with the default-mode system (mean *r* = −0.082, *p* < 0.001 after Bonferroni correction for multiple comparisons, *t*(1206) = −27, 95% CI: [−0.088, −0.076]; Fig. 1c right). Using a multilevel model, we found that these correlations are significantly different (*β* = −0.12469, *p* < 0.0001, *t*(1769) = −37.4, *SE* = 0.0033272, *n* = 2414).

To further unpack these findings, we also considered the relation between these two subnetworks and the dorsal attention system, which has been described as antagonistic to the default-mode system in working memory tasks^9,10,19^. Consistent with this account, we found that the activity of subnetwork (A) was negatively correlated with the activity of the dorsal attention system (mean *r* = −0.014, *p* < 0.001 after Bonferroni correction for multiple comparisons, *t*(1206) = −7.22, 95% CI: [−0.18, −0.11]; Supplementary Fig. 9A, right), while the activity of subnetwork (B) was positively correlated with the activity of the dorsal attention system (mean *r* = 0.12, *p* < 0.001 after Bonferroni correction for multiple comparisons, *t*(1206) = 39, 95% CI: [0.11, 0.12]; Supplementary Fig. 9A, left). Using a multilevel model, we found that these correlations are significantly different (*β* = 0.13057, *p* < 0.0001, *t*(1769) = 46.8, *SE* = 0.0027885, *n* = 2414).

Given that subnetwork (B) is functionally connected to the dorsal attention system, it is interesting to ask whether subnetwork (B) should be considered a formal part of the dorsal attention system during this task. Likewise, it is interesting to ask whether subnetwork (A) should be considered part of the default-mode system during this task. To address this question, we calculated the difference between (i) the strength of functional connections within subnetwork (B) and (ii) the strength of functional connections between subnetwork (B) and the dorsal attention system. We calculated this difference 10,000 times, bootstrapping over subjects, and found that regions within subnetwork (B) were more strongly connected to each other than to the dorsal attention system (*p* < 0.001). Performing an analogous experiment, we found that regions within subnetwork (A) were more strongly connected to each other than to the default-mode system (*p* < 0.001). In other words, subnetwork (A) appears to be functionally distinct from the default-mode system, and subnetwork (B) appears to be functionally distinct from the dorsal attention system. For the results of complementary analyses identifying and characterizing genetic differences between subnetworks, see Supplementary Note 7.

To address the final proposition in our model, we sought to determine whether increased subnetwork (A) activity would lead to a stronger positive functional connection between the frontoparietal and default-mode systems, while increased subnetwork (B) activity would lead to a stronger negative functional connection between the two systems. Because the summation of the subnetwork timeseries should reflect the complete frontoparietal timeseries, we also reasoned that when subnetwork (A) is more active (higher amplitude) relative to subnetwork (B), the frontoparietal signal would be more similar to the dynamics of subnetwork (A) than to the dynamics of subnetwork (B). To test these expectations, we began by quantifying subnetwork activity using the root mean square (RMS) of the subnetwork timeseries. We then used a single robust linear model to explain the strength of the functional connection between the default-mode and frontoparietal systems by a linear combination of the activity of subnetwork (A) and the activity of subnetwork (B). Consistent with our hypothesis, we found that an increase in subnetwork (A) activity corresponded to a stronger positive functional connection between the two systems (estimate of regression coefficient *β* = 0.006535, 95% CI: (0.00519, 0.00788), *p* < 0.001, *t*(1204) = 9.54, Fig. 1d), while an increase in subnetwork (B) activity corresponded to a stronger negative functional connection between the two systems (estimate of regression coefficient *β* = −0.0112, 95% CI: (−0.01273, −0.0097), *p* < 0.001, *t*(1204) = −14.3, Fig. 1e). In a complementary analysis, we used a single robust linear model to explain the strength of the functional connection between the dorsal attention and the frontoparietal systems by a linear combination of the activity of subnetwork (A) and the activity of subnetwork (B). We found that an increase in subnetwork (A) activity corresponded to a stronger negative functional connection between the two systems (*β* = −0.00553, *p* < 0.001, *t*(1204) = −8.717, 95% CI: (−0.00677, −0.00486)), while an increase in subnetwork (B) activity corresponded to a stronger positive functional connection between the two systems (*β* = 0.00989, *p* < 0.001, *t*(1204) = 13.7, 95% CI: (0.00848, 0.011316)).

The results of the three tests described above serve to validate the formal structure of our model. Next, we turned to an assessment of the relevance of this complex dynamical system for behavior. Specifically, we had observed previously that behavioral performance decreases as the correlation between the frontoparietal and default-mode systems increases. Here, we seek to explain that observation using the activity of the two subnetworks. Because subnetwork (A) is positively related to frontoparietal–default-mode connectivity, which is itself negatively related to behavioral performance, we would expect subnetwork (A) activity to be negatively related to behavioral performance. Conversely, because subnetwork (B) is related negatively to frontoparietal–default-mode connectivity, which is itself negatively related to behavioral performance, we would expect subnetwork (B) activity to be positively related to behavioral performance. To probe these relationships, we fit a single robust multilevel model with behavioral performance as the dependent variable, and subnetwork (A) and subnetwork (B) activity as independent variables. As expected, we found that subnetwork (A) activity is negatively related to behavioral performance (*β* = −0.00271, *p* < 0.001, *t*(1194) = −3.83, 95% CI: (−0.0041, −0.00132)), and subnetwork (B) activity is positively related to behavioral performance (*β* = 0.00273, *p* < 0.001, *t*(1194) = 3.40, 95% CI: (0.00115, 0.00430)). Finally, we asked whether the functional connection between the frontoparietal and the default-mode system accounts for the variance in behavioral performance even after regressing out the effects of the activity of subnetwork (A)? In other words, is the relationship between functional connectivity and behavioral performance just a natural consequence of subnetwork (A) activity, or does it provide additional explanatory power for the behavior? To address this question, we fit a robust linear mixed-effect model using behavioral performance as the dependent variable, and both subnetwork (A) activity and frontoparietal–default-mode connectivity as independent variables, and we found that the behavior–connectivity relationship remained significant (estimate of regression coefficient for connectivity: *β* = −0.254, *p* < 0.001, *t*(1194) = −9.84, 95% CI: (−0.3047, −0.2034), estimate of regression coefficient for subnetwork (A) activity: *β* = −0.00056, *p* = 0.004, *t*(1194) = −2.66, 95% CI: (−0.00098, −0.00014)). This result suggests that the frontoparietal–default-mode functional connection explains additional behavioral variance beyond what is explained by the activity of subnetwork (A) alone.

### The structural role of the frontoparietal subnetworks

We next turn to an examination of what, if any, neuroanatomical support exists for the subnetwork-driven dynamics espoused in the previous section. Recent advances in network control theory have posited that changes in the activation of single brain regions can induce a propagation of activity along white matter tracts to affect distributed circuit behavior in a predictable fashion^15,20^. Here, we test this notion within the specific confines of our experiment, asking: does the structural connectivity of frontoparietal subnetworks constrain how activity propagates to neighboring areas, thereby modulating the coupling between the frontoparietal and default-mode systems? We hypothesize that subnetwork (A) is more structurally connected to the default-mode system, while subnetwork (B) is more structurally connected to the dorsal attention system. This hypothesis is based on evidence that a higher number of white matter tracts between two regions can support stronger functional connectivity between them^21^, allowing subnetwork (A) to strongly couple to the default-mode system, and subnetwork (B) to strongly couple to the dorsal attention system.

**Fig. 2:**
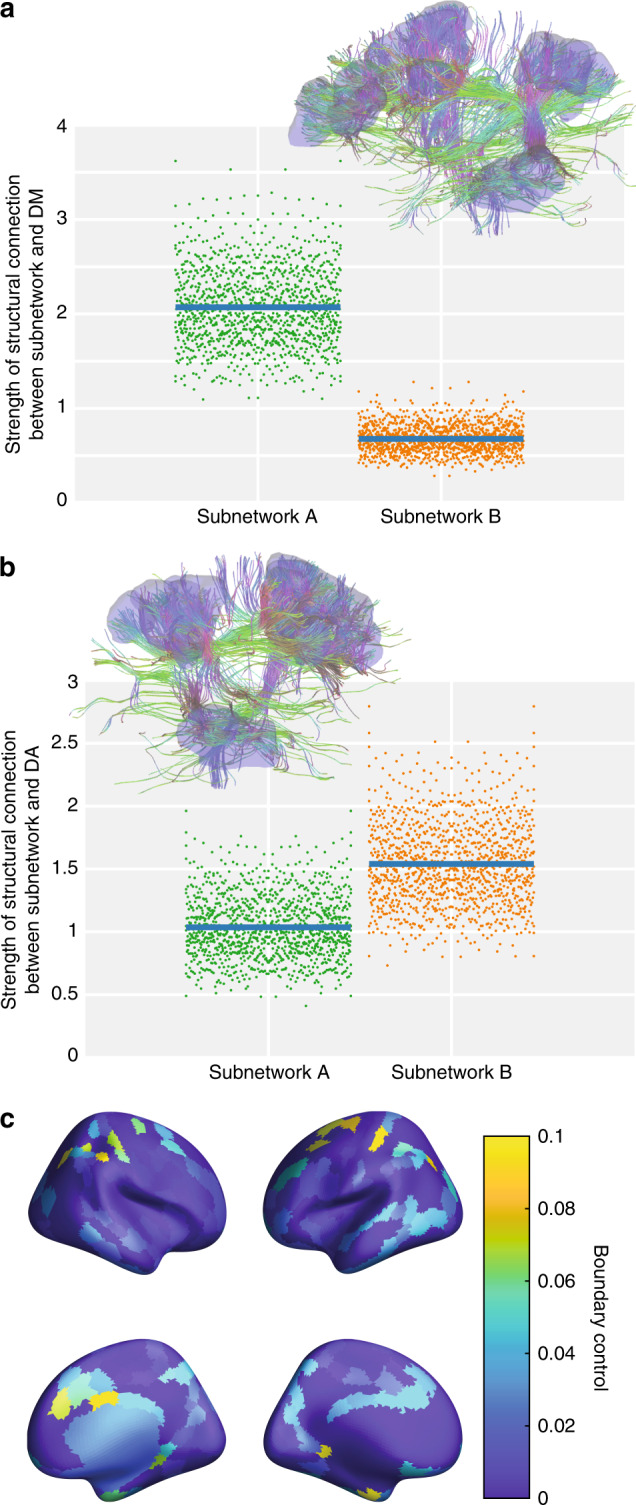
White matter connectivity of the frontoparietal subnetworks. **a** Subnetwork (A) is more strongly structurally connected to the default-mode system (mean = 2.07, 95% CI: (2.05, 2.09)) than is subnetwork (B) (mean = 0.676, 95% CI: (0.668, 0.685)). **b** Subnetwork (A) is less strongly structurally connected to the dorsal attention system (mean = 1.03, 95% CI: (1.02, 1.04)) than is subnetwork (B) (mean = 1.54, 95% CI: (1.52, 1.56)). Note that for visualization purposes, the data were not visually adjusted to account for two points coming from each subject, whereas the reported statistics do take this into account. The insets of panels (**a**) and (**b**) display the white matter fibers emanating from subnetworks (A) and (B), respectively. **c** The anatomical distribution of boundary control calculated with respect to the default mode and dorsal attention systems is overrepresented in the frontoparietal system in comparison with a nonparametric permutation-based null model.

We tested this hypothesis by calculating the strength of the structural connectivity between subnetworks and systems using diffusion imaging tractography (see “Methods”). Consistent with our hypothesis, we found that subnetwork (A) is more strongly connected to the default-mode system (mean = 2.07, *p* < 0.001 after Bonferroni correction for multiple comparisons, *t*(1206) = 173, 95% CI: (2.04, 2.09), Fig. 2a, left) than is subnetwork (B) (mean = 0.676, *p* < 0.001 after Bonferroni correction for multiple comparisons, *t*(1206) = 156, 95% CI: (0.668, 0.685), Fig. 2a right). Using a multilevel model, we found that these correlations were significantly different (*β* = −0.0013939, *p* < 0.0001, *t*(1769) = −174.8, *SE* = 7.9759 × 10^−6^, *n* = 2414). Similarly, we found that subnetwork (B) is more strongly connected to the dorsal attention system (mean = 1.54, *p* < 0.001, *t*(1206) = 162, 95% CI: (1.52, 1.56), Fig. 2b, left) than is subnetwork (A) (mean = 1.03, *p* < 0.001 after Bonferroni correction for multiple comparisons, *t*(1206) = 146, 95% CI: (1.02, 1.04), Fig. 2b, right). Using a multilevel model, we found that these correlations were significantly different (*β* = 0.00050413, *p* < 0.0001 after Bonferroni correction for multiple comparisons, *t*(1769) = 99.3, *SE* = 5.0721 × 10^−6^, *n* = 2414).

In addition to the direct structural connections considered above, indirect structural connections have been shown to contribute meaningfully to functional connectivity, although to a smaller extent than direct structural connections^22^. We therefore examined the effect of indirect structural connections in Supplementary Note 8.

Last, we considered whether these results may be partially driven by spurious connections, and therefore assessed the reliability of the results over a range of thresholds. The particular method that we employ was previously designed to probe the robustness of results to the elimination of weak connections^23^. Specifically, we evaluated the effects reported in Fig. 2a, b when eliminating the weakest 50%, 60%, 70%, 80%, and 90% of connections. We found that the significance of the results shown in Fig. 2a, b did not change at any of these threshold levels. Interestingly, we found that the variance in strength of a particular structural connection is related to the mean strength of that connection (*r* = 0.9191, *p* < 0.0001), suggesting that the results in Fig. 2a, b are not driven by noisy connections with high variance.

Collectively, these data suggest that the frontoparietal subnetworks might be well positioned in the structural connectome to mediate coupling between the default-mode and dorsal attention areas, a coupling that is negatively correlated with performance (Supplementary Fig. 15). To more directly test this notion, we calculated the regional boundary control (Eq. 2) with respect to the default-mode and dorsal attention systems (Fig. 2c). Of the 20 regions for which boundary control exceeded the 95th percentile, 9 were located in the frontoparietal system. To evaluate statistical significance, we compared these results to those of a nonparametric null model, in which we randomly permute the association between boundary control values and brain regions. We found that the probability that 9 or more of the top 20 regions fell within the frontoparietal system was significant with respect to the null model (*p* = 0.0019). In summary, these data suggest that the frontoparietal system is structurally positioned to effectively control the coupling between the default mode and dorsal attention systems.

### A dynamical model relating system activity and connectivity

**Fig. 3:**
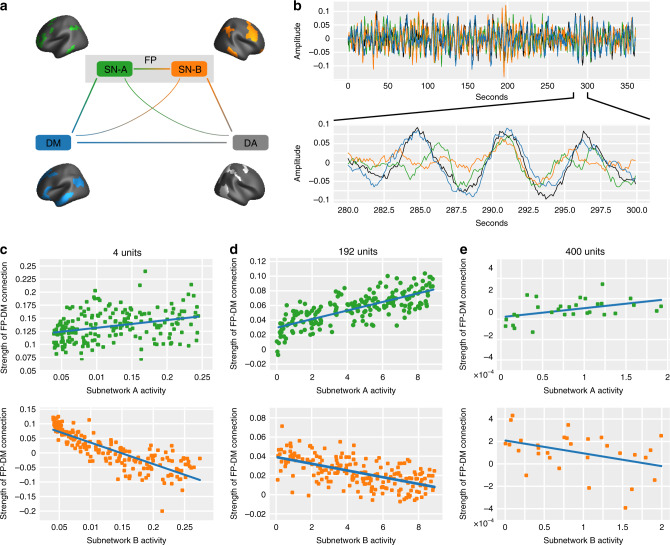
A simplified model for studying the relationships between brain system activity and connectivity. **a** We constructed a dynamical model of a 4-, 192-, and 400-oscillator representation of either the four systems (default mode, DM;  frontoparietal subnetworks, SN-A & SN-B; and dorsal attention, DA) of interest (4- and 192-oscillator systems), or of the entire brain (400-oscillator system). Here, we show a representation of this oscillator network consisting of the frontoparietal subnetwork (A), the frontoparietal subnetwork (B), the default mode system, and the dorsal attention system. The remaining oscillators are not shown here representing all other regions of the brain used in the 400-oscillator model. **b** The network dynamics were integrated out to a total time of 6 min, consistent with the length of the empirical n-back scan. **c** Using four oscillators, increasing the amplitude of subnetwork (A) activity, while keeping all others equal, caused an increase in the functional connectivity between the frontoparietal and default mode units (Pearson correlation coefficient between unit timeseries: *r* = 0.396, *p* = 0.001), while increasing the amplitude of subnetwork (B) activity caused a decrease in the functional connectivity between the frontoparietal and default mode units (Pearson correlation coefficient between unit timeseries: *r* = −0.866, *p* < 0.0001). **d** Using 192 oscillators, increasing the amplitude of subnetwork (A) activity caused an increase in the functional connectivity between the frontoparietal and default mode units (*r* = 0.753, *p* = 0.001), while increasing the amplitude of subnetwork (B) activity caused a decrease in the functional connectivity between the frontoparietal and default mode units (*r* = −0.599, *p* < 0.0001). **e** Using 400 oscillators, increasing the amplitude of subnetwork (A) activity caused an increase in the functional connectivity between the frontoparietal and default mode units (*r* = 0.453, *p* = 0.011), while increasing the amplitude of subnetwork (B) activity caused a decrease in the functional connectivity between the frontoparietal and default mode units (*r* = −0.394, *p* = 0.031). Note that panel (**e**) contains the results of 30 simulations, in contrast to the 200 simulations depicted in panels **c** and **d**; this difference is due to the marked increase in computational burden in the 400-oscillator model.

In the previous sections, we found evidence consistent with (but not proving) the causal notion that increased activity of subnetwork (A) drives stronger coupling between the frontoparietal and default-mode systems, while increased activity of subnetwork (B) drives anticorrelation between the two systems. While it is difficult to prove the validity of such a causal model in healthy human participants, we can gather additional supportive evidence from in silico experiments^24^ exercising a formal computational model of the dynamical system. Specifically, we implemented a coarse-grained dynamical model of a four-node network (Fig. 3a), where each unit in the network represented one of the following four human brain systems: default-mode, dorsal attention, and frontoparietal subnetworks A and B. The strength of system-level structural connections was estimated by averaging the individual edge strengths of node–node structural connections (Fig. 3a). In order to model the oscillatory behavior of brain system activity, we consider each unit in the network to be an oscillator, with dynamics described by the normal form of a Hopf bifurcation, and with frequencies randomly sampled from an empirically measured distribution (see “Dynamical network model” for details). We coupled the four nodes according to the mean weight of the structural connections between them, as estimated from diffusion tensor imaging tractography, averaged across subjects (see “Methods”). The model has two free parameters: (i) the global coupling parameter, which tunes the general capacity for synchronization, and (ii) the bifurcation parameter of each oscillator, which tunes the amplitude of the oscillator timeseries (Supplementary Fig. 12). Following a broad parameter sweep, we selected parameter values to ensure a realistic dynamical regime between a state of no synchrony and a state of complete synchrony among all oscillators (see “Methods” and Fig. 3b).

Next, we implemented the model to probe the relation between subnetwork activity and connectivity, focusing initially on the connectivity between the frontoparietal and default-mode systems. In agreement with our empirical results, we found that increasing the amplitude of subnetwork (A) activity increased the correlation between the frontoparietal and default-mode unit timeseries (Pearson’s correlation coefficient *r* = 0.396, *p* = 0.001, Fig. 3c, top). Importantly, to ensure that our results are not trivially explained by global changes in coupling, we subtract the overall mean system coupling from the strength of the functional connection between the frontoparietal and default-mode timeseries. Similarly, and again in agreement with our empirical results, we found that increasing the amplitude of subnetwork (B) activity decreased the correlation between the frontoparietal and default-mode unit timeseries (Pearson’s correlation coefficient *r* = −0.866, *p* < 0.00001, Fig. 3c, bottom). To determine the relationship between the subnetworks, we initialized the dynamic model 200 times, each time calculating the correlation between the resulting timeseries for subnetwork (A) and subnetwork (B). We found that the two timeseries were anticorrelated in 197 of the 200 runs, suggesting that in this model, these two systems tend to be anticorrelated (*p* = 0.015). To assess the reliability of these results, we performed the same numerical experiments for a range of coupling and bifurcation parameter values, across which the effects remained robust (Supplementary Fig. 13).

Although we chose to begin with a simplified model setup that allowed us to focus specifically on the interactions between the four brain systems of interest in this study, it is crucial to be aware that (1) each of these systems is actually composed of several individual brain regions, and (2) these systems are part of a larger whole-brain network composed of many other areas. In light of these facts, it is important to determine whether the relationships we found using the simple 4-unit system can be reproduced in more complex and realistic network representations. To examine this question, we implemented two complementary approaches: a 192- and a 400-unit network. In the 192-unit formulation, we still considered only the four systems/subsystems examined in this study, but rather than collapsing each system into a single node, we represented each of the four systems at the resolution of the 400-region Schaefer parcellation. Specifically, subnetwork (A) was represented by 30 nodes, subnetwork (B) was represented by 31 nodes, the default-mode system was represented by 79 nodes, and the dorsal attention system was represented by 52 nodes. To determine the effect of subnetwork (A) activity on the functional connection between the frontoparietal and default-mode systems, we initialized all non-subnetwork (A) oscillators at the same activity, and varied the initial activity of subnetwork (A) oscillators by tuning their bifurcation parameters as in the simpler version of the model. This process was then repeated, but for the case of varying subnetwork (B) activity. We found that we were able to reproduce both of the above findings relating subnetwork A and B activity to frontoparietal–default-mode functional coupling at this finer scale (Fig. 3d). Notably, the two above models assume that the four systems of interest exist in isolation. To address this simplification, we built one final model: to the 192-unit model, we added 208 other regions, hence constructing a complete cortical representation. In this 400-unit model, we were also able to recover the same relationships (Fig. 3e).

Next, we examined the complementary relation between subnetwork activity and the connectivity between the frontoparietal and dorsal attention systems. Again, consistent with our empirical results, we found that increasing subnetwork (A) activity decreased the correlation between the frontoparietal and dorsal attention unit timeseries (Pearson’s correlation coefficient *r* = −0.216, *p* = 0.002; Supplementary Fig. 14A, right), and increasing subnetwork (B) activity increased the correlation between the frontoparietal and dorsal attention unit timeseries (Pearson’s correlation coefficient *r* = 0.538, *p* < 0.0001; Supplementary Fig. 14A, left). Importantly, we were able to reproduce these results in the 192-unit model (Supplementary Fig. 14B).

Lending support to the notion that these dynamics may emerge from the structural coupling of the oscillators, we attempted to reproduce the results from Fig. 3a, b after eliminating all structural connections between oscillators. We found that these alterations eliminated both the relationship between subnetwork activity and frontoparietal–default-mode connection strength for both subnetwork (A) (*r* = 0.0707, *p* = 0.301) and subnetwork (B) (*r* = −0.0707, *p* = 0.753). Collectively, this pattern of results offers tentative support for a candidate mechanism, in which subnetwork activity tunes functional connectivity. It is not known how these results, originating from our in silico model implementation, may relate to in vivo brain function. Our findings serve to support the conceptual framework of our subnetwork hypothesis, but the putative causal structure described above may not necessarily translate outside of this computational model.

## Discussion

Our findings suggest that, during engagement with a working memory task, the frontoparietal system (and not the default-mode system) may partially govern the functional connection between the frontoparietal and default-mode systems. Relevantly, the frontoparietal system is known to flexibly alter its functional connections dynamically according to current task demands^25^, perhaps controlling the strength of connectivity between cognitive systems^4^. In order to support such a broad range of cognitive states, it has been suggested that the frontoparietal system may be composed of subnetworks, where each subnetwork subserves a specific cognitive state^17,26^. Mechanistically, one possible explanation for how the frontoparietal system could drive itself to be either correlated or anticorrelated with the default-mode system is if it is composed of two disjoint subnetworks, where subnetwork (A) displays activity that is correlated with the activity of the default-mode system, and where subnetwork (B) displays activity that is anticorrelated with the activity of the default-mode system. Using an unsupervised clustering algorithm informed by an explicit model of network architecture, we demonstrated that the frontoparietal system is decomposable into two subnetworks with distinct patterns of functional connections.

Prior work by Dixon and colleagues has found a similar division of the frontoparietal system^17^. The authors employed a hierarchical clustering method to establish a data-driven partition of the frontoparietal system into two components, the first component being more functionally connected to the default mode, similar to our subnetwork (A), and the second component being more functionally connected to the dorsal attention system, similar to our subnetwork (B). Our findings critically extend these prior observations by providing evidence that by supporting competition between the two systems, subnetwork (B) may be critical to working memory performance, while subnetwork (A) may be less involved in working memory, and more closely linked with introspective processes. Importantly, while functional divisions of the frontoparietal system have previously been put forth, these divisions have not yet been used to motivate a mechanistic understanding of competition between brain networks. In our mechanistic framework, this finding provides a possible explanation for how the frontoparietal system may independently and partially govern the functional connection between the frontoparietal and default-mode systems, enabling competition between the two systems (which itself is correlated with individual differences in performance). Thus, behavioral performance is related to competition, and competition is driven by the activity of frontoparietal subnetwork (B), which is anticorrelated with the default-mode system. Broadly, these results indicate that specific network interactions subserving certain types of human cognition may be driven by just the dynamics of a subnetwork of one (rather than both) of the involved networks.

In addition to their functional distinguishability, we also demonstrated that these two subnetworks displayed distinct patterns of gene coexpression. In line with this observation, it is interesting to note that prior work has suggested that cortical regions responsible for different cognitive functions can express different genes^27^, and that gene coexpression provides a partial explanation for patterns of functional connectivity^28^. In agreement with these findings, our results demonstrate more similar patterns of gene expression within subnetworks than between subnetworks, an effect that cannot be explained by interregional distance. Notable prior work has also suggested a link between structural connectivity and gene expression^29^, also supported by our finding that the two genetically dissimilar subnetworks display differing patterns of white matter connectivity. It is interesting to speculate that these genetic dissimilarities are partially responsible for the subnetworks’ differential capacity to tune the coupling between the frontoparietal and default-mode systems via relative changes in activity amplitudes. In summary, the frontoparietal system is decomposable into two discrete, nonoverlapping functional subnetworks: subnetwork (A) is functionally aligned with the default-mode system, and subnetwork (B) is functionally opposed to the default-mode system. This arrangement of two subsystems in functional opposition suggests a possible mechanism by which the functional connection between the frontoparietal and default-mode systems can be modulated: namely, the alteration of relative activity levels across subsystems. Furthermore, individual differences in this functional opposition between the two subnetworks may underlie individual differences in working memory performance.

Above, we established that frontoparietal subnetwork (B) is in functional competition with the default-mode system, and the activity of this subnetwork may drive competition between the frontoparietal and default-mode systems. Next, to expand our mechanistic understanding, we sought to explore why subnework (B) is a driver of competition, while subnetwork (A) is a driver of cooperation. Motivated by prior work demonstrating that structural and functional connectivity share topographic similarities^21,30^, we extend our study to multimodal data to better understand the potential structural drivers constraining the manner in which activity in frontoparietal subnetworks impinges on functional coupling in other systems. We found that subnetwork (A) had fewer structural connections to the dorsal attention system than it had to the default-mode system, and also than subnetwork (B) had to the dorsal attention system. Similarly, we found that subnetwork (B) had fewer structural connections to the default-mode system than it had to the dorsal attention system, and also than subnetwork (A) had to the default-mode system. Mechanistically, these data suggest that structural connectivity may play a role in constraining the functional dynamics of the two subnetworks. To better understand this role, we draw on tools from network control theory, which provides an additional mechanistic framework to link network structure to functional network dynamics^15,31,32^. Regions with high levels of boundary control are theoretically posited to have the capacity to steer the brain to different states by coupling and decoupling cognitive systems^15^. Our results demonstrate that the frontoparietal subnetworks are situated within the white matter architecture in a manner that can drive the system’s coupling with the dorsal attention and default-mode systems. The effective activity of this hub of network control may be cognitively advantageous by, during externally directed tasks, buffering the attentional systems from the internally directed processes of the default-mode system, and likewise during internally directed tasks buffering the default-mode system from external stimuli. In summary, the structural coupling of the subnetworks mirrors the functional coupling of the subnetworks, suggesting that large-scale white matter tracts might support the functional connectivity observed during this task. Furthermore, subnetwork (B) may be an efficient driver of competition between the frontoparietal and default-mode systems due to its position in the white matter scaffolding.

We proposed a model wherein the competitive coupling between the frontoparietal and default-mode systems is modulated by activity levels in the oscillatory dynamics of two frontoparietal subnetworks. To test the validity of this putative mechanism, we employed a simplified dynamical model of brain system activity using oscillators coupled by empirically determined structural connectivity^33–35^. This model allowed us to directly test our hypotheses by allowing us to alter subnetwork amplitude, and subsequently to observe the resulting synchronization (functional connectivity) between the frontoparietal and default-mode systems. The results from these simulated experiments agree with our hypotheses, and demonstrate that the modulation of subnetwork amplitude governs intersystem coupling in a way that matches the empirical discoveries. This finding provides one possible mechanistic explanation for our results: the frontoparietal system may mediate the effective functional coupling between the dorsal attention and default-mode systems.

It has been proposed that the frontoparietal system is functionally interposed between the dorsal attention and default-mode systems, altering their functional coupling in a task-specific manner. In particular, during externally directed tasks, the frontoparietal system may engage with the dorsal attention system and disengage with the default-mode system^36^. Conversely, during internally directed tasks, the frontoparietal system disengages with the dorsal attention system, and engages with the default-mode system^19^. Together, these complementary processes are thought to effectively segregate external stimuli from internal trains of thought during tasks that require more focus on one of the two. Our results demonstrate that this complementarity can be reproduced in an oscillator model via the differential activation and deactivation of oscillator groups representing two frontoparietal subnetworks. Indeed, our results suggest that increasing the oscillation amplitude of oscillators representing subnetwork (A) activity synchronizes the frontoparietal oscillator group with the default-mode oscillator group, and increasing the oscillation amplitude of oscillators representing subnetwork (B) desynchronizes the frontoparietal oscillator group with the default-mode oscillator group.

These results suggest a putatively causal relationship between activity and connectivity in silico; how these putative results may relate to in vivo brain function requires further investigation. The frontoparietal system is thought to flexibly reconfigure functional connections with the dorsal attention and default-mode systems in a task-dependent manner^9^, and our results offer evidence for a mechanism by which this flexible reconfiguration may be achieved. In summary, we found correlative results in fMRI data, suggesting that subnetwork activities are related to the strength of the functional connection between the frontoparietal and default-mode systems. Specifically, increased activity in subnetwork (A) is correlated with decreased strength of the frontoparietal–default-mode connection; in contrast, increased activity in subnetwork (B) is correlated with increased strength of the frontoparietal–default-mode connection. Modeling these functional systems as collections of oscillators, we observed that increasing the activity of subnetwork (A) in silico decreases the strength of the frontoparietal–default-mode connection, and increasing the activity of subnetwork (B) in silico increases the strength of the frontoparietal–default-mode connection. Although the oscillator ensemble model is quite simple relative to the true system, the results from the model lend support to a causal interpretation of the above correlative findings.

Our study provides evidence for a mechanism by which the dynamics of the frontoparietal system may drive working memory performance. Two distinct subnetworks within the frontoparietal system play a role in modulating the functional coupling between the frontoparietal and default-mode systems during the performance of an n-back working memory task, which may help buffer the externally directed attentional system from internal trains of thought, and lead to improved behavioral performance. We found that the position of the two subnetworks within the white matter scaffolding constrains the distinct function of each: one is structurally tied to the dorsal attention system (and drives the frontoparietal system into competition with the default-mode system), and the other is structurally tied to the default-mode system (and drives the frontoparietal system into cooperation with the default-mode system). We extend these descriptive observations by building a computational model instantiating and demonstrating the putative mechanism, and we bolster our findings with corroborating differences in gene expression in the two subnetworks. Together, our findings contribute to a holistic view of working memory by linking activity, functional connectivity, structural connectivity, and gene expression, and present one way of understanding how these four modes work in concert to support cognitive processes necessary for working memory.

## Methods

### Imaging data acquisition and preprocessing

For each subject in the Human Connectome Project (HCP) S900 release^37^, we extracted the task-based functional magnetic resonance imaging data acquired during the performance of the n-back working memory task, a resting-state functional magnetic resonance imaging scan, a high-resolution anatomical scan, and a diffusion tensor imaging scan. In this release, 644 subjects contained all four data types, all four resting-state scans, and BedpostX diffusion data. Participants included 346 females, and the full sample had a mean age (std) of 28.6 (3.68) years. No additional exclusion criteria were applied. All analyses were performed in accordance with the relevant ethical regulations of the WU-Minn HCP Consortium Open Access Data Use Terms. Informed consent was obtained in writing from all participants.

The acquisition parameters for each data type are as follows. The parameters for the acquisition of the high-resolution structural scan were TR = 2400 ms, TE = 2.14 ms, TI = 1000 ms, flip angle = 8°, FOV = 224 × 224 mm, voxel size = 0.7-mm isotropic, BW = 210 Hz/Px, and acquisition time = 7:40 min. Functional magnetic resonance images were collected during both rest and task with the following parameters: TR = 720 ms, TE = 33.1 ms, flip angle = 52°, FOV = 208 × 180 mm, matrix = 104 × 90, slice thickness = 2.0 mm, number of slices = 72 (2.0-mm isotropic), multifactor band = 8, and echo spacing = 0.58 ms. Diffusion tensor images were collected with the following parameters: TR = 5520 ms, TE = 89.5 ms, flip angle = 78°, refocusing flip angle = 160°, FOV = 210 × 180, matrix = 168 × 144, slice thickness = 1.25 mm, number of slices = 111 (1.25-mm isotropic), multiband factor = 3, echo spacing = 0.78 ms, and *b* vaues = 1000, 2000, and 3000 s/mm^2^.

We focused our analyses on data acquired during the n-back task, due to its reliable recruitment of the executive system^38^. The working memory task was presented at two different levels of difficulty: 0-back and 2-back. For both levels, subjects were presented with a stream of images taken from the following four categories: faces, places, tools, or body parts. The latter images presented body parts that were whole (nonmutilated); no images contained nudity. In the 0-back condition, subjects were meant to respond positively during every image presentation. In the 2-back condition, subjects were meant to respond positively if the present image was identical to the image presented two images previously. The task was divided into two runs, each run being composed of eight task and two fixation blocks^39^. Fixation blocks lasted for 15 s each. Each task block consisted of ten trials, where a stimulus was presented for 2 s, followed by a 500-ms ITI (2.5 s in total per trial)^39^. Each block begins with a 2.5-s cue, indicating the 0-back or 2-back condition. During the 0-back condition, working memory loads are minimal. Within each run, half of the task blocks used the 2-back paradigm, and half used the 0-back paradigm. In addition, within a run, each stimulus type (images from a single category) was presented in a different block. To estimate behavioral performance, we calculated the accuracy of responses across all image categories separately for 0-back and 2-back conditions. We chose to focus on accuracy due to its interpretability^4^. However, we also considered d-prime^38,40^, and demonstrate that our main results hold when using this metric in place of accuracy (see Supplementary Note 3).

For both resting-state and task-based functional connectivity, CompCor, with five principal components from the ventricles and white matter masks, was used to regress out nuisance signals from the timeseries. In addition, the 12 detrended motion estimates provided by the Human Connectome Project were regressed out from the timeseries. The mean global signal was removed, and then timeseries were band-pass filtered from 0.009 to 0.08 Hz. Finally, frames with greater than 0.2-mm framewise displacement or a derivative root mean square (DVARS) above 75 were removed as outliers. Sessions composed of greater than 50% outlier frames were not further analyzed.

We chose to regress out the global signal from the timeseries because it has been shown to remove motion signal and global scanner noise. We also note that the mathematics of global signal regression does not necessitate a specific spatial distribution of negative correlations^41^, and our claims regard the relative strengths of connectivity between networks rather than their sign. Moreover, the processing pipeline used here has been suggested to be ideal for removing false relations between connectivity and behavior^42^. Finally, we note that our main results hold when using wavelet coherence as a measure of functional connectivity (see Supplementary Note 5); this measure is bounded between 0 and 1.

For the diffusion imaging, the Human Connectome Project applied intensity normalization across runs, the TOPUP algorithm for EPI distortion correction, the EDDY algorithm for eddy current and motion correction, gradient nonlinearity correction, calculation of gradient *b*-value/*b*-vector deviation, and registration of mean *b*0 to native volume T1w with FLIRT. BBR+bbregister and transformation of diffusion data, gradient deviation, and gradient directions to 1.25-mm structural space were also applied. The brain mask is based on the FreeSurfer segmentation. The BedpostX (Bayesian Estimation of Diffusion Parameters Obtained using Sampling Techniques) output was then calculated, where the “X” stands for modeling crossing fibers. Markov Chain Monte Carlo sampling was used to build probability distributions on diffusion parameters at each voxel. The process creates all of the files necessary for running probabilistic tractography. Using the Freesurfer recon-all data computed by the Human Connectome Project, the fsaverage5 space cortical parcellation was registered to the subject’s native cortical white matter surface, and then transformed to the subject’s native diffusion volume space. From these data, we derived seeds and targets for probabilistic tractography, which we ran with the FSL probtrackx2 algorithm using 1000 streams initiated from each voxel in a given parcel.

We parcellated the brain into 400 discrete and nonoverlapping regions of interest using the Schaefer atlas (fslr32k surface)^43^. Notably, the Schaefer atlas was originally validated in the same HCP data that we study here, and it yields a functional demarcation of both the default-mode and the frontoparietal systems. Of course, other functionally defined atlases exist, but they are less ideal for our purposes for several reasons: the Power atlas^44^ does not provide full cortical coverage, and the Gordon^45^ and Brainnetome^46^ atlases are of lower spatial resolution, including 333 and 246 regions, respectively. The Schaefer atlas provides an assignment of each region to one of 17 putative cognitive systems: two visual, two somatomotor, two dorsal attention, two salience/ventral attention, one limbic, three frontoparietal, three default-mode, and one temporoparietal system. To ensure that the granularity of the data was consistent with the granularity of our hypotheses, we collapsed these 17 systems into eight systems by combining individual systems that belonged to the same cognitive system, that is, we combined the two visual systems into a single system, the two somatomotor systems into a single system, the two dorsal attention systems into a single system, the two salience systems into a single system, the three frontoparietal systems into a single system, and the three default-mode systems into a single system.

### Analysis of functional magnetic resonance imaging data

**Fig. 4:**
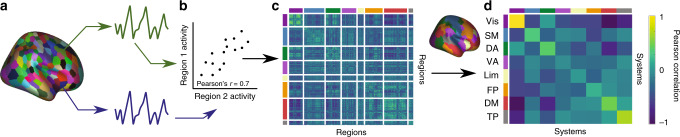
Methodological schematic. **a** fMRI BOLD images from 644 subjects in the HCP S900 release were segmented into 400 regions to extract regional mean timeseries. **b** We assessed the functional connectivity between each pair of regions by calculating the Pearson correlation coefficient between the timeseries of region *i* and the timeseries of region *j*. **c** We encoded all pairwise functional connectivity estimates in a functional connectivity matrix, which offers a formal representation of the network model under study. Each region was assigned to one of eight intrinsic functional systems defined a priori. **d** From this assignment, we constructed a system-by-system functional connectivity matrix where each element indicates the average strength of all functional connections for region pairs, in which one region of the pair is located in system *i* and the other region of the pair is located in system *j*. Systems are color-coded and ordered from left to right (and from top to bottom) as follows: visual (Vis), somatomotor (SM), dorsal attention (DA), salience or ventral attention (VA), limbic (Lim), frontoparietal (FP), default-mode (DM), and temporoparietal (TP).

We used the preprocessed data to construct functional connectivity matrices reflecting functional interactions between regions and systems. Specifically, we extracted processed timeseries from each of the 400 regions in the Schaefer atlas (Fig. 4a). Next, we calculated the Pearson correlation coefficient between each pair of regional timeseries (Fig. 4b). We chose to use the Pearson correlation to represent functional connectivity due to its widespread use in the neuroimaging literature, as well as its ease of interpretability^47^, but we also demonstrate robustness of our results to other measures of functional connectivity in Supplementary Note 5. We collated all interregional estimates of functional connectivity into a single 400 × 400 connectivity matrix, ***C***_**f**_ (Fig. 4c), which we then treated as the formal encoding of brain function^48^. To be explicit, in this network representation, regions are represented by network nodes, and functional connections are represented by weighted edges, where the weight of the edge between node *i* and node *j* is given by the Pearson correlation coefficient between the timeseries of region *i* and the timeseries of region *j*. Finally, we averaged the estimates of functional connectivity within systems, and between pairs of systems, to construct a system-by-system connectivity matrix (Fig. 4d).

To partition the frontoparietal system into two functionally disjoint groups, we employed the weighted stochastic block model, which is a powerful community detection method. This method is complementary to the more widely used modularity maximization methods^49^, but is noted to have increased flexibility and sensitivity to a more diverse set of network architectures^50–53^. Briefly, the weighted stochastic block model is a generative model that places each of the *N* nodes of network ***C***_**f**_ into one of *K* communities. This placement is accomplished by finding a network partition ${\mathbf{z}} \in {\cal{Z}}^{N\! \times\! 1}$ where **z**_*i*_ ∈ {1, 2, …, *K*}, **z**_*i*_ denotes the membership of node *i*, and ***C***_**f**_ is an *N* × *N* matrix encoding pairwise functional connections. Assuming that network edge weights are normally distributed, following Refs. ^54,51^, the generative model takes the following form:

1 $$P\left( {{\boldsymbol{C}}_{\mathbf{f}}\left| {{{\mathbf{{z}}}},{\boldsymbol{\mu}} ,{\boldsymbol{\sigma}} ^2} \right.} \right) = \mathop {\prod}\limits_{i = 1}^N {\mathop {\prod}\limits_{j = 1}^N {{\mathrm{exp}}\left( {{\boldsymbol{C}}_{{\mathbf{f}},ij} \cdot \frac{{{\boldsymbol{\mu}} _{{{\mathbf{{z}}}}_{i}{{\mathbf{{z}}}}_{{j}}}}}{{{\boldsymbol{\sigma}} _{{{\mathbf{{z}}}}_{{i}} {{\mathbf{{z}}}}_{{j}}}^2}} - \frac{{{\boldsymbol{C}}_{{\mathbf{f}},ij}^2}}{{2{\boldsymbol{\sigma}} _{{{\mathbf{{z}}}}_{{i}} {{\mathbf{{z}}}}_{{j}}}^2}} - \frac{{{\boldsymbol{\mu}} _{{{\mathbf{{z}}}}_{{i}}{{\mathbf{{z}}}}_{{j}}}^2}}{{{\boldsymbol{\sigma}} _{{{\mathbf{{z}}}}_{{i}}{{\mathbf{{z}}}}_{{j}}}^2}}} \right)}}.$$

Here we have introduced model parameters ${\boldsymbol{\mu}} \in {\cal{R}}^{K \times\! K}$ and ${\boldsymbol{\sigma}} ^2 \in {\cal{R}}^{K \times\! K}$, where ${\boldsymbol{\mu}}_{{\mathbf{{z}}}_{{i}}{{\mathbf{{z}}}}_{{j}}}$ and ${\boldsymbol{\sigma}} _{{\mathbf{{z}}}_{i}{\mathbf{{z}}}_j}^2$ parameterize the weights of normally distributed connections between community **z**_*i*_ and community **z**_*j*_, and ***C***_**f**__*,ij*_ denotes the *ij-*th element of the network ***C***_**f**_. Furthermore, $P\left( {{\boldsymbol{C}}_{\mathbf{f}}\left| {{\mathbf{{z}}},{\boldsymbol{\mu}} ,{\boldsymbol{\sigma}} ^2} \right.} \right)$ is the probability of generating the observed network ***C***_**f**_ given the parameters. This model is fit to ***C***_**f**_ in order to estimate the parameters **z**, ***μ***, and ***σ***^2^. We fit the model using MATLAB code provided in Ref. ^54^ and freely available at http://tuvalu.santafe.edu/aaronc/wsbm/.

For each subject, we fit the weighted stochastic block model to the *m* × *m* subgraph of the functional connectivity matrix representing functional connections between the *m* = 61 regions of the frontoparietal system. We selected *K* = 2 a priori due to prior evidence that the frontoparietal system can be separated into two distinct components^17^. The implementation generated a single maximum likelihood partition of regions into functional communities for each subject. Then, we pooled partitions across subjects, and used a consensus similarity method^18^ to identify a single partition that is most similar to all others, where similarity is quantified by the *z* score of the Rand coefficient^55^. To assess the statistical significance of this partition, we performed a nonparametric permutation test. Specifically, for each subject, we calculated the log-likelihood of the weighted stochastic block model fitting the final consensus partition to their individual functional network. As a null, we calculated the log-likelihood of the weighted stochastic block model fitting a random permutation of the final consensus partition to their individual network. We assessed the difference between the true and null data using a multilevel model (see “Statistical analysis”).

### Analysis of diffusion tensor imaging data

After performing probabilistic tractography, we applied the same 400-region Schaefer atlas. Next, we calculated the proportion of streams seeded in a voxel in one region that reached another region. We chose to use the proportion of streamlines to represent structural connectivity due to the inhomogeneity of the region sizes. We collated all interregional estimates of structural connectivity into a single 400 × 400 connectivity matrix, ***C***_**s**_, which we then treated as the formal encoding of a network representation of brain structure^48^. Similar to the model of brain function, in this structural network representation, regions are represented by network nodes, and structural connections are represented by weighted edges, where the weight of the edge between node *i* and node *j* is given by the proportion of streams seeded at region *i* that reach region *j*. Finally, we averaged the estimates of structural connectivity within systems, and between pairs of systems, to construct a system-by-system connectivity matrix, akin to the one that we constructed from the functional data. In our analysis of these data, all structural matrices were normalized by the total weight of all connections^23^.

We posited that structural connections between systems would play an important role in the functional coupling between the frontoparietal and default-mode systems. Specifically, we hypothesized that the formal nature of that role was one of boundary controllability, more commonly studied in the field of control and dynamical systems theory^56^. Boundary control is a quantifiable metric describing the notion that the topological location of a region within a structural network partially governs that region’s influence on the function of modules or communities in the network^15,31^. Intuitively, if region *i* has strong structural connections to regions *j* and *l*, then the activity of region *i* influences the functional connection between regions *j* and *l*. Boundary control assesses the positioning of a region between two other regions, and can be calculated for region *i* with respect to its control over regions *j* and *l* as follows:

2 $${\mathrm{BC}}(i) = \left\{ {\begin{array}{*{20}{c}} {1 - \left( {\frac{{{{\mathbf{k}}}_i(j)}}{{{{\mathbf{k}}}_i}}} \right)^2 - \left( {\frac{{{{\mathbf{k}}}_i(l)}}{{{{\mathbf{k}}}_i}}} \right)^2} & {{\mathrm{for}}\,{{\mathbf{k}}}_i(j) + {{\mathbf{k}}}_i(l) = {{\mathbf{k}}}_i} \\ {\left( {\frac{{{{\mathbf{k}}}_i(j)}}{{{{\mathbf{k}}}_i}}} \right)^2 + \left( {\frac{{{{\mathbf{k}}}_i(l)}}{{{{\mathbf{k}}}_i}}} \right)^2} & {{\mathrm{for}}\,{{\mathbf{k}}}_i(j) + {{\mathbf{k}}}_i(l)\, <\, {{\mathbf{k}}}_i} \end{array}} \right .$$

Here, **k**_*i*_, the degree of region *i*, is the sum of all region *i*’s structural connections. The variables **k**_*i*_ (*j*) and **k**_*i*_ (*l*) are the strength of region *i*’s structural connections to regions *j* and *l*, respectively. A region with high boundary control is predicted to more effectively modulate the functional connection between region *j* and region *l*, although boundary control does not assess whether that modulation will increase or decrease the connection strength. In summary, a region *i* with high boundary control with respect to regions *j* and *l*—a feature of region *i*’s structural coupling—is theoretically expected to effectively alter the strength of functional coupling between regions *j* and *l*.

### Gene coexpression analysis

To determine whether the two subnetworks that we identified in the frontoparietal system displayed distinct patterns of gene expression, we used gene expression data from six postmortem brains available from the Allen Brain Institute. We focused our analyses on 16,699 genes that had previously been identified as relevant for brain function^28^. Data for these specific genes were available in 338 of the 400 brain regions. We assigned the anatomical location of each probe to one of 338 parcels defined a priori. For each parcel and each gene, we calculated the mean expression of that gene across all probes, after subtracting the mean expression of each probe for that gene^57^. Collectively, these calculations generated a data matrix of size 338 (parcels) by 16,699 (mean expression across probes in that parcel for a given gene).

Gene coexpression between parcel *i* and parcel *j* is measured by the Pearson correlation coefficient *r* between gene expression values of parcel *i* and gene expression values of parcel *j*. To assess subnetwork specificity of gene coexpression, we first drew a bootstrap sample of 16,699 genes and constructed a 338 × 338 gene coexpression matrix. Second, from this coexpression matrix, we calculated the mean gene coexpression both within and between subnetworks. Between-subnetwork coexpression is the mean of the gene coexpression values for pairs of nodes for which one node in the pair is located in subnetwork (A) and the other node in the pair is located in subnetwork (B). Third, we calculated the ratio of within- to between-network gene coexpression, where a ratio >1 indicates greater within-subnetwork coexpression than between-subnetwork coexpression. Finally, we compute the difference between this ratio and a ratio expected in a nonparametric null model. To construct the null model, we randomly permuted subnetwork membership 1000 times, and recomputed coexpression ratios for each permutation. The expected ratio of the null model is equal to the mean of the null distribution. Each random sample of 16,699 genes generates a single index, indicating whether the ratio of within- to between-network gene coexpression is greater or less than what we expect from the above-described null model. Next, we repeat the above process 10,000 times, selecting a different random sample of 16,699 genes each time, generating a distribution of differences, indicating whether the true ratio is larger than the mean of the null model ratios. In total, this algorithm provides us with a distribution of 10,000 indices, indicating whether the ratio of within- to between-network gene coexpression is greater or less than we would expect. To complete our statistical analysis, from this distribution, we compute the probability of the difference being less than 0. For additional analyses controlling for the distance between regions, see the Supplementary Information.

### Dynamical network model

As motivated more fully in the “Results” section, we propose that two subnetworks of the frontoparietal system work in functional opposition to either couple or decouple the frontoparietal system from the default-mode system. Specifically, we suggest that the functional connection between the frontoparietal and default-mode systems is governed by the relative activation amplitudes of the two frontoparietal subnetworks. To further probe these relationships, we built a coarse-grained network in which each unit represented a particular brain system, and simulated system dynamics with a canonical coupled oscillator model. More specifically, network activity was modeled by the normal form of a supercritical Hopf bifurcation (also referred to as the Stuart–Landau model), which describes the transition between a state of low activity and a state of oscillatory dynamics^58,59^. We chose this model because (i) it permits the independent manipulation of oscillator amplitudes (Supplementary Fig. 12), allowing us to further investigate the empirically observed relationships between activity and connectivity, and (ii) it is often used to model large-scale brain activity^33–35,60,61^. Following Refs. ^33,34^, the local dynamics of the *j-*th unit are given by the following equation:

3 $$\frac{{{\mathrm{d}}{\mathbf{u}}_j}}{{{\mathrm{d}}t}} = {\mathbf{u}}_j[{\mathbf{a}}_j + i{\boldsymbol{\omega}} _j - |{\mathbf{u}}_j|^2] + \xi {\boldsymbol{\eta}} _j(t),$$

where ${\mathbf{u}}_j = {\boldsymbol{\rho}} _je^{i\theta _j} = {{\mathbf{x}}}_j + i{{\mathbf{y}}}_j$, ***η***_*j*_ is drawn from a normal distribution to add Gaussian noise to the system, and *ξ* scales the noise. In Eq. (3), the term **a**_*j*_ is commonly called the *bifurcation parameter*. When **a**_*j*_ < 0, the system goes to a low-activity fixed point and when **a**_*j*_ > 0, the system obeys a stable limit-cycle solution with angular frequency ***ω***_*j*_ and signal amplitude governed by **a**_*j*_.

Following Refs. ^33,34^, we model a network of interacting components by separating the system into its real and imaginary parts, and we link different components via the following set of coupled differential equations:

4 $$\frac{{{\mathrm{d}}{{\mathbf{x}}}_j}}{{{\mathrm{d}}t}} = \left[ {{{\mathbf{a}}}_j - {{\mathbf{x}}}_j^2 - {{\mathbf{y}}}_j^2} \right]{{\mathbf{x}}}_j - {\boldsymbol{\omega}} _j{{\mathbf{y}}}_j + G\mathop {\sum }\limits_{i = 1}^{n_o} {{\mathbf{D}}}_{ij}\big( {{{\mathbf{x}}}_i - {{\mathbf{x}}}_j} \big) + \xi {\boldsymbol{\eta}} _j(t),$$

and

5 $$\frac{{{\mathrm{d}}{{\mathbf{y}}}_j}}{{{\mathrm{d}}t}} = \left[ {{{\mathbf{a}}}_j - {{\mathbf{x}}}_j^2 - {{\mathbf{y}}}_j^2} \right]{{\mathbf{y}}}_j + {\boldsymbol{\omega}} _j{{\mathbf{x}}}_j + G\mathop {\sum}\limits_{i = 1}^{n_o} {{{\mathbf{D}}}_{ij}\big( {{{\mathbf{y}}}_i - {{\mathbf{y}}}_j} \big) + \xi {\boldsymbol{\eta}} _j\left( t \right)} .$$

Here, *G* is the coupling strength. As suggested by Ref. ^34^, we set *ξ* = 0.02, and we took *x*_*j*_ as the oscillatory signal of interest. To estimate the frequency parameters, we empirically calculated the peak frequency for each subject of each oscillator during the resting state, fit a normal distribution to the peak-frequency values, and drew from the normal distribution. Similarly, to establish **D**_*ij*_, the coupling of the network nodes, we calculated the mean structural connectivity estimated from diffusion tractography between system *i* and system *j* across all subjects. We integrated the equations using a time step of 0.01 s for 6 min, which was the approximate length of the task scans. For a discussion of the effect of these empirically derived parameters on model results, see Supplementary Note 10.

To estimate reasonable values for the coupling and bifurcation parameters, we conducted a parameter sweep (Supplementary Fig. 11), and computed both the root mean square of the timeseries, and the synchrony among all oscillators using the Kuramoto order parameter, defined at time point *t* as

6 $$R\left( t \right) = \frac{1}{{n_{\mathrm{o}}}}\left| {\mathop {\sum}\limits_{j = 1}^{n_{\mathrm{o}}} {{\mathrm{e}}^{i\phi _j\left( t \right)}} } \right|$$

where *ϕ*_*j*_ (*t*) is the instantaneous phase of oscillator *j* at time *t*, and *n*_o_ is the total number of oscillators, which in our case is four (default-mode system, dorsal attention system, frontoparietal subnetwork (A), and frontoparietal subnetwork (B)). To get a summary statistic for the entire timeseries, we took the mean of *R* across time (Supplementary Fig. 11). The instantaneous phase was computed by taking the Hilbert transform of the unfiltered timeseries. As shown in Supplementary Fig. 11, we focus on a subset of the parameter space, in which the units exhibit oscillatory behavior. Notably, outside this subset, the units do not display pronounced oscillatory behavior, but instead reach fixed points where their dynamics do not change over time.

For our baseline working point, we selected *a* = −0.075 and *G* = 0.1 for all units, where the Kuramoto order parameter has an intermediate value, signifying a realistic dynamical regime between a state of no synchrony and a state of complete synchrony among all oscillators. Furthermore, at this working point, the root mean square of the timeseries is higher than the noise level (Supplementary Fig. 11). For each point in our grid defined by *a* and *G*, we averaged results from ten initializations of the system. During each system initialization, we drew new frequency parameters from the fitted normal distributions described above. Importantly, the bifurcation parameter of each oscillator is linearly related to the root mean square of the time series values of that node (Supplementary Fig. 12B).

### Statistical analysis

At several points throughout the study, we calculate the statistical difference between outcome variables of the two subnetworks. To that end, we initially take a parametric approach, and then we confirm all of our findings using a nonparametric permutation-based approach. In all visualizations of statistical relationships, subject effects have been regressed out from the dependent variable. Last, we perform several pairs of statistical tests in which we estimate the effect of a dependent variable on two independent variables in two separate tests. All such tests pass Bonferroni correction for multiple comparisons.

In testing our hypotheses, we often asked questions of the following form: does the strength of the connection between subnetwork (A) and the default-mode system differ from the strength of the connection between subnetwork (B) and the default-mode system? For questions of this form, we used a multilevel model where each outcome variable (e.g., a measurement of connection strength) has attributes encoding subnetwork membership, task run, and subject identity. The multilevel model framework^62^ accounts for the nested nature of the data (multiple scans nested within the subject). We specified the model as

7 $${\mathrm{Outcome}}\,{\mathrm{Variable}}_{it} = {\cal{B}}_{0i} + {\cal{B}}_{1i}{\mathrm{SubNetwork}}_{it} + e_{it},$$

where OutcomeVariable_*it*_ is the outcome variable (i.e., connection strength) for person *i* on run *t*; ${\cal{B}}_{0i}$ indicates the level of the outcome in subnetwork (A); ${\cal{B}}_{1i}$ indicates differences in the level of outcome associated with subnetwork (B) versus subnetwork (A); *e*_*it*_ are residuals.

Person-specific intercepts (from Level 1) were specified (at Level 2) as

8 $${\cal{B}}_{0i} = \gamma _{00} + u_{0i},$$

and

9 $${\cal{B}}_{1i} = \gamma _{10},$$

where *γ* denotes a sample-level parameter and *u*_0*i*_ denotes residual between-person differences that may be correlated, but are uncorrelated with *e*_*it*_. Intuitively, this constitutes a random-effect model, where we allow person-specific random intercepts (specified by Eq. 8) in the main model (Eq. 7). The multilevel model was fit with *lme* in R using maximum likelihood estimation. In the case of many outliers, we treat our data with robust models, rather than standard linear models. Robust models downweight points of data, where the most outlying points are downweighted most severely. Specifically, we implement robust multilevel models using *robustlmm* in R^63^. We note in the text whenever a robust multilevel model is used.

Unless otherwise noted, we use a repeated measures correlation when examining the association between two continuous variables^64^. The repeated measures correlation accounts for nonindependence among observations (due to multiple runs per subject) by using a form of analysis of covariance (ANCOVA) to adjust for between-person variance. The model is specified as

10 $${\mathrm{Measure}}1_{it} = \overline {{\mathrm{Measure}}2} _i + {\mathrm{Subject}}_i + c({\mathrm{Measure}}2_i) + e_{it},$$

where Measure1_*it*_ is the value of variable one for subject *i* during measurement occasion *t*, $\overline {{\mathrm{Measure}}2} _i$ is the mean value of the second variable in the *i-*th participant, Subject_*i*_ is a unique identifier for each participant, and *c*(Measure2_*i*_) is the covariate for the *i-*th participant and is equal to ${\cal{B}}({\mathrm{Measure}}2_{it} - \overline {{\mathrm{Measure}}2} _i)$, where ${\cal{B}}$ is the slope coefficient of the covariate. Like a Pearson correlation coefficient (*r*), the repeated measures correlation (*rrm*) is bounded between −1 and 1, and represents the strength of the linear association between two variables. The repeated measures correlation was estimated using the *rmcorr* package in R^64^.

In addition to the multilevel linear model, we employ a complementary permutation-based approach. We begin with vectors $Y_1 \in {\cal{R}}^{1 \times 2n}$, $Y_2 \in {\cal{R}}^{1 \times 2n}$, and $S \in {\cal{R}}^{1 \times 2n}$, where *n* is the number of subjects, and each subject has participated in two scanning sessions. We would like to test whether the difference in the means of *Y*_1_ and *Y*_2_ is greater than expected in an appropriate statistical null model. Our null hypothesis is that there is no difference between the means of *Y*_1_ and *Y*_2_. We therefore use a nonparametric permutation-based null model in which we randomly permute the assignment of vector elements in *Y*_1_ and *Y*_2_ to two new vectors $Y\prime _1$ and $Y\prime _2$. Specifically, we individually visit each row *i* of the 2*n* rows, where each row *i* corresponds to a specific session for a specific subject, and at random, we either (1) swap the *i-*th element between *Y*_1_ and *Y*_2_, or (2) do not swap the *i-*th element. This procedure results in a random permutation of the vector assignment for each session for each subject. After the permutation process, we calculate the mean difference between pairwise elements of the permuted vectors. In order to generate a null distribution, we repeat the above permutation process 10,000 times. We determine a *p* value for the true effect by calculating the proportion of null differences that are greater than the observed difference.

### Methodological limitations

Several methodological limitations are pertinent to this work. In this paper, the frontoparietal system was divided into two subnetworks, creating group-level subnetworks. First, it should be noted that the subnetworks could also have been studied at the level of single individuals, although such granularity could hamper the ability to draw group-level conclusions. Second, these subnetworks were defined using a community detection algorithm based on a specific generative model. There exist several methods to find communities within networks, each with its own set of underlying assumptions. As a result, careful consideration must be taken when selecting the appropriate method of community detection. The flexibility of the WSBM makes it the most reasonable choice, given the data used here.

While the employed dynamical model indeed recovered empirical results, and allowed us to postulate mechanistic explanations, it is important to point out some of its methodological limitations. One limitation of our model revolves around the scale at which it operates. In particular, because our main empirical findings concern system-level dynamics—rather than dynamics at the scale of individual neurons or parcels—we assumed, for simplicity, that the different units in our computational model represented different brain regions. This coarse-grained approach is beneficial for a number of reasons. For example, it simplifies our analysis, and allowed us to focus explicitly on macroscopic, region-level drivers of various results, and therefore directly compares output from the model to the corresponding empirical findings. However, although informed by experimental data, it is critical to acknowledge that such a setup is a great simplification of the true system, and allows little room for understanding how observations at the level of brain systems arise from interactions between more microscopic structural components.

Along the same vein, the dynamics of each brain system were described inherently phenomenologically, via a Hopf bifurcation model^59^, which has been utilized in studies concerned with the interplay between network structure and dynamics in general^65,66^, and also in computational studies on brain network dynamics more specifically^33–35^. In particular, such dynamics indeed capture the oscillatory nature of observed brain system activity, but do not embody a biophysically precise description of neuronal activity. Therefore, the model cannot attempt to describe the emergence of brain system dynamics from dynamical processes on smaller scales. In the future, one may choose to employ an alternative method of modeling, such as dynamic causal modeling^67^, which would allow for probing other complementary and nuanced aspects of the proposed model. Although in this investigation, we have chosen, as a first step, to employ a canonical model with few parameters that favors simplicity and interpretability, building and analyzing more realistic and detailed, multiscale models, is an important area of ongoing research.

### Reporting summary

Further information on research design is available in the Nature Research Reporting Summary linked to this article.

## Supplementary information

Supplementary Information

Reporting Summary

## Data Availability

The diffusion MRI, functional MRI, and behavioral data that support the findings of this study are available in the Human Connectome Project (humanconnectomeproject.org). Gene expression data are available from the Allen Brain Institute (alleninstitute.org). A reporting summary for this article is available as a Supplementary Information file.

## Code availability

The computer code used to generate simulations and conduct analyses of the data contained in this study is available upon request.
