# Supplementary material for: Multimodal network dynamics underpinning working memory
Source: Nat Commun. 2020 Jun 15;11:3035. doi: 10.1038/s41467-020-15541-0 (PMC7295998; doi:10.1038/s41467-020-15541-0)
Supplement: Supplementary file 1 — Supplementary Information [file 41467_2020_15541_MOESM1_ESM.pdf]

Supplement to:

"Multimodal network dynamics underpinning working memory"

Andrew C. Murphy[1,2], Maxwell A. Bertolero[1], Lia Papadopoulos[3], David M. Lydon-Staley[1], and Danielle S. Bassett[1,3,4,5,6,7,*]

[1]Department of Bioengineering, School of Engineering & Applied Science, University of Pennsylvania, Philadelphia, PA 19104, USA

[2]Perelman School of Medicine, University of Pennsylvania, Philadelphia, PA 19104, USA

[3]Department of Physics & Astronomy, School of Arts & Sciences, University of Pennsylvania, Philadelphia, PA 19104, USA

[4]Department of Neurology, Perelman School of Medicine, University of Pennsylvania, Philadelphia, PA 19104, USA

[5]Department of Electrical & Systems Engineering, School of Engineering & Applied Science, University of Pennsylvania, Philadelphia, PA 19104, USA

[6]Department of Psychiatry, Perelman School of Medicine, University of Pennsylvania, Philadelphia, PA 19104, USA

[7]Santa Fe Institute, Santa Fe, NM USA 87501, USA

[*]To whom correspondence should be addressed: dsb@seas.upenn.edu

Supplementary Note 1 : Connectivity between default mode and frontoparietal systems tracks working memory performance.

We first sought to replicate the finding that functional connectivity between the default mode and frontoparietal systems tracks executive function performance, and show that this holds on this 2-back working memory task. For each participant, we calculated the average functional connectivity between all relevant pairs of brain regions, where one region of the pair was located in the default mode system and the other region of the pair was located in the frontoparietal system. We then estimated the relationship between behavioral accuracy and between-system strength. We found that the strength of the between-system connection was negatively correlated with performance on the task across subjects (repeated measures correlation, $r = -0.4731$, $p < 0.001$, $DF = 558$; Supplementary Figure 1A).

To assess task-specificity, we repeated the analysis using the imaging and performance data from the 0-back condition. During this condition, minimal working memory load exists. We found no significant relationship between behavioral performance and the strength of the connection between the default mode and frontoparietal systems (repeated measures correlation; $r = -0.0293$, $p = 0.487$, $DF = 562$; Supplementary Figure 1B-C). To assess the robustness of the connectivity-behavior relationship, we used an alternate behavioral measure (see Supplementary note 3 and Supplementary Figure 3), an alternate network parcellation (see Supplementary Note 4 and Supplementary Figure 4, Supplementary Figure 5), and an alternate measure of functional connectivity (see Supplementary note 5 and Supplementary Figure 6). The results of these additional analyses serve to confirm our main findings. While in the above analysis, we related on-line behavioral performance with concurrent imaging, the HCP dataset has a rich repository of off-line behavioral metrics as well. We examined two off-line measures of executive function: the NIH Toolbox Dimensional Change Cart Sort Test (DCCS), which assesses cognitive flexibility, and the NIH Toolbox Flanker Inhibitory Control and Attention Test (Flanker), which assesses attention and inhibitory control. We found that in-scanner n-back performance was related to off-line performance on the DCCS ($r = 0.31$, $p < 0.0001$) and on the Flanker ($r = 0.26$, $p < 0.0001$). Further, we found that FPS activity related to off-line behavioral performance on the DCCS ($r = 0.163$, $p < 0.0001$) and on the Flanker ($r = 0.11$, $p = 0.0004$).

In a remarkably diverse range of tasks, the frontoparietal system tends to increase in activity while

the default mode system tends to decrease in activity [1, 2]. A common and intuitive interpretation of these findings is that the two systems exist in *competition*, consistently displaying anti-correlated dynamics with one another. From a cognitive perspective, it has been proposed that such competition is indicative of opposing goals of the two systems [3, 4], and may enable toggling between the cognitive states supported by each system [5]. Importantly, the degree of competition between the frontoparietal and default mode systems has been linked to individual differences in performance on a range of tasks including the categorization of emotion in faces [6], the flanker task [1], and several tasks taxing human reasoning abilities [7]. Interestingly, attenuation of the competition between these two systems is related to decreases in executive function occurring in older age [8].

The nature of the functional connection between the frontoparietal and default mode systems appears to be flexible and task-dependent [9, 10]. Externally directed tasks tend to elicit competition while internally-directed tasks tend to elicit *cooperation* [11, 12, 13]. For example, the two systems show competitive dynamics during visuo-spatial planning, and cooperative dynamics during autobiographical planning [14]. Likewise, the two systems show cooperative dynamics during a finger-tapping task, and competitive dynamics during a movie watching task [15]. Similarly, the two systems have been found to show cooperative dynamics during a goal-directed simulation task requiring imagination [16], as well as during the production of internal trains of thought [17]. It has been proposed that the default mode system is critically involved in self-referential tasks [18], and that coupling to the frontoparietal networks allows attention to be directed internally to these tasks, rather than to external stimuli [17, 18]. Our study extends this prior literature by demonstrating the existence of competitive dynamics between the two systems during the performance of an n-back working memory task, where the degree of competition is related to *individual differences* in behavioral performance.

While the notion that interactions between brain networks may influence behavior has been demonstrated in many different contexts [1, 19, 20], a critical unaddressed question remains: How do these functional interactions emerge from system dynamics? Here, we address this question in the context of competition between the frontoparietal and default mode systems during working memory tasks. Intuitively, the functional relationship between the frontoparietal and default mode systems depends explicitly on the activity dynamics of those two systems. To begin forming a mechanistic understanding of competition, we asked the question: is competition between the frontoparietal and

default mode systems dependent equally on the activities of the two systems, or is competition explained by the activity of one system more than the other? In other words, may one system drive competition more strongly than the other?

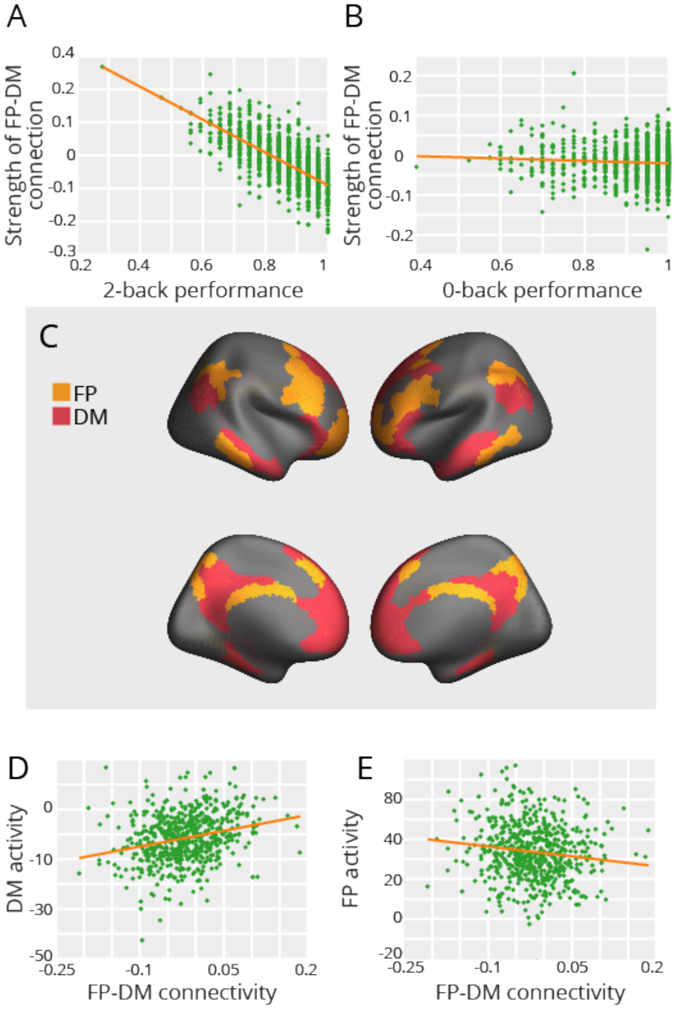

Supplementary Figure 1: **Functional connectivity and activity in the frontoparietal system relate to working memory performance.** *(A)* We found that the strength of the connection between the frontoparietal and default mode systems is negatively correlated with performance on the 2-back working memory task (repeated measures correlation, $r = -0.4731$, $p < 0.001$, $DF = 558$. *(B)* During the 0-back task, we found no significant relationship between performance and the strength of the connection between the frontoparietal and default mode systems (repeated measures correlation; $r = -0.0293$, $p = 0.487$, $DF = 562$). *(C)* Anatomical location of regions within the frontoparietal and default mode systems, displayed on the cortical surface. *(D)* Activity of the default mode system was positively correlated with the strength of the functional connection between the frontoparietal and default mode systems (Pearson correlation; $r = 0.2565$, $p = 1.282 \times 10^{-10}$. *(E)* Activity of the frontoparietal system was negatively correlated with the strength of the functional connection between the frontoparietal and default mode systems (Pearson correlation; $r = -0.1305$, $p = 0.0012$).

Supplementary Note 2 : The activity of the frontoparietal system is correlated with the strength of the functional connection between the frontoparietal and default mode systems.

After observing a statistical relation between working memory performance and the functional connectivity between the default mode and frontoparietal systems, we sought to better understand its potential drivers. We began by testing the hypothesis that system activity drives inter-system connectivity. We defined regional activity as the parameters from a GLM fit to the regional BOLD magnitude during the 2-back task (for details of the GLM, see Supplementary Note 9). To assess the activity of a system, we averaged those GLM parameters across all regions in the frontoparietal system or across all regions in the default mode system. We observed that the frontoparietal system activity was positive on average (one-sample t-test; mean = 22.5, $p < 0.001$, $t(800) = 42$, whereas the default mode system activity was negative on average (one-sample t-test; mean = -6.7, $p < 0.001$, $t(800) = -14.8$). Furthermore, we found that the difference in activity between the two systems was significant using a multilevel model ($\beta = -7.4501$, $p < 0.0001$, $t(1769) = -35.7$, $SE = 0.20847$).

In assessing the relationship between system activity and inter-system connectivity, we found that the activity of the default mode system is positively related to the strength of the connection between the default mode and frontoparietal systems (Pearson correlation; $r = 0.2565$, $p = 1.282 \times 10^{-10}$; Supplementary Figure 1D). In contrast, we found that the activity of the frontoparietal system was negatively correlated with the strength of this connection (Pearson correlation; $r = -0.1305$, $p = 0.0012$; Supplementary Figure 1E). Given that frontoparietal activity is correlated with the strength of the inter-system connectivity, and that the inter-system connectivity is correlated with behavioral performance, it stands to reason that the frontoparietal activity should also be correlated with behavioral performance. Indeed we found that the activity of the frontoparietal system is positively related to behavioral performance (Pearson correlation; $r = 0.1024$, $p = 0.0117$; Supplementary Figure 2A), while the activity of the default mode system is unrelated to behavioral performance (Pearson correlation; $r = 0.0524$, $p = 0.1978$; Supplementary Figure 2B).

To identify putative drivers of competition between the frontoparietal and default mode systems, we first related system activity to inter-system connectivity. We began by investigating the relationship between default mode system activity and competition. Interestingly, we do not observe a

relationship between default mode activity and individual differences in performance (Supplementary Figure 2B), consistent with a previous study of group-averaged n-back performance in patients with chronic pain and healthy matched controls [21]. From a mechanistic perspective, this finding suggests that the activity of the default mode system may not be a strong driver of competition between the frontoparietal and default mode systems. This notion is bolstered by findings suggesting that a threshold may exist, below which working memory performance is unaffected by any further decreases in default mode activity [21], which may eliminate the default mode system as a driver of competition. In line with this observation, prior studies have shown that the activity of the default mode decreases [22, 23], consistent with a suppression of mind-wandering and other self-referential processes that can hamper external goal-directed behavior [24]. Thus, cognitive engagement with these tasks may drive the default mode network activity below the hypothesized threshold. Agreeing with these findings, our results demonstrate that during n-back performance, the default mode system is less active on average than the frontoparietal system.

In contrast to default mode activity, frontoparietal activity directly tracked individual differences in behavior, with higher frontoparietal activation being associated with greater task accuracy (Supplementary Figure 2A). Mechanistically, this finding suggests that, out of the two systems, frontoparietal may be the major driver of competition. Lending support to this notion, the frontoparietal system is thought to act as a flexible hub during task performance [25], altering its connectivity with the default mode system in a task-dependent fashion [26, 14]. Indeed, it has been proposed that the frontoparietal system is functionally (and structurally) interposed between the default mode and dorsal attention systems [27], modulating their coupling in a task specific way [5]. Further, Hellyer and colleagues proposed that increased frontoparietal activity promotes persistent stable states, whereas increased default mode activity may allow for transitions between cognitive states, which in turn would be undesirable during tasks requiring directed and fixed attention [9]. Thus, an active frontoparietal system may also help to maintain a persistent competitive relationship with the default mode, aiding in improved behavioral performance. Collectively, these results suggest that – in the specific case of interactions between the frontoparietal and default mode systems during working memory – the competitive network interaction may be driven by activity of the frontoparietal system, but not the default mode system. Broadly, these results indicate that specific network interactions subserving certain types of human cognition *may* be driven by the dynamics of just one (rather than both) of

the involved networks.

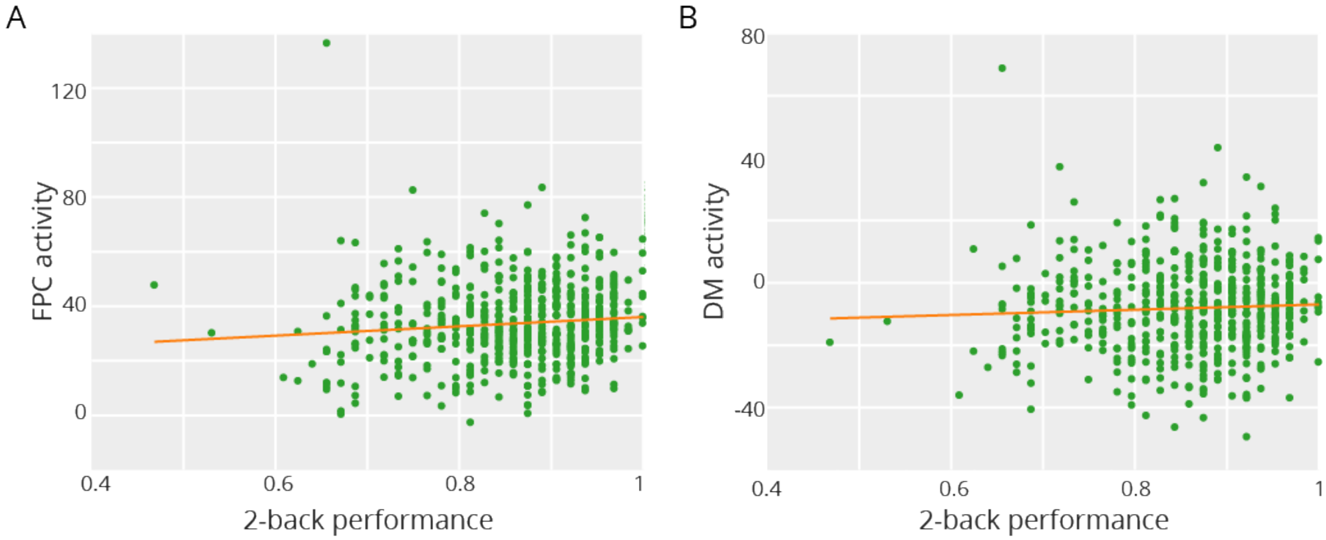

Supplementary Figure 2: **Frontoparietal activity correlates with working memory performance.** During the 2-back working memory task, we found that the activity of the frontoparietal system is related to behavioral performance (see panel *(A)*; Pearson correlation; $r = 0.1024$, $p = 0.0117$), while the activity of the default mode system is unrelated to behavioral performance (see panel *(B)*; Pearson correlation; $r = 0.0524$, $p = 0.1978$).

Supplementary Note 3 : Alternate behavioral measure

In the main text, behavioral performance on the n-back working memory task was defined as the total accuracy, measured by the ratio of correct responses to total responses. To probe the robustness of our results, we examined an alternate behavioral metric given by the sensitivity index (d'). The sensitivity index quantifies a subject's ability to discriminate between previously seen and novel stimuli [28] and is sometimes used as an alternative to accuracy in the study of behavioral performance on working memory tasks [29]. The d' metric and accuracy are correlated with one another during the 0-back task (repeated measures correlation; $r = 0.89$, $p < 0.001$, $DF = 562$) and during the 2-back task (repeated measures correlation; $r = 0.852$, $p < 0.001$, $DF = 558$). Using this alternate metric, we found that the relationship between behavior and the functional coupling between the frontoparietal and default mode systems remained unchanged. Furthermore, using this alternate metric, we found that the relationship between behavior and the activity of the frontoparietal system remained unchanged, as did the relationship between behavior and the activity of the default mode system (Supplementary Figure 3).

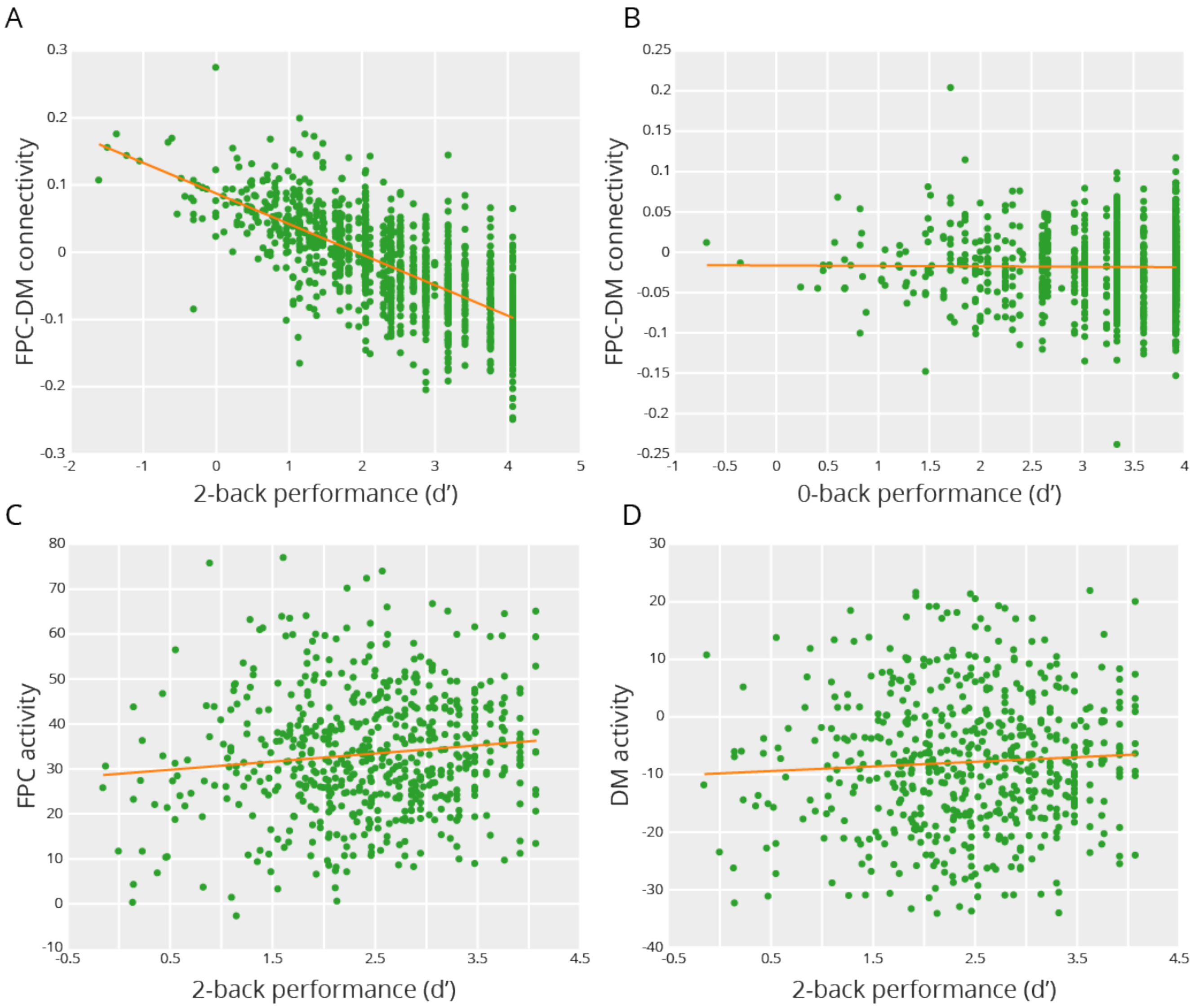

Supplementary Figure 3: **The relationship of connectivity and activity to behavior (d').** Using the alternate behavioral metric of d', the relationship of behavior to both connectivity and activity remained unchanged. Specifically, the functional coupling between the frontoparietal and default mode systems during the 2-back task is negatively correlated with performance (see panel *(A)*; repeated measures correlation; $r = -0.4738$, $p < 0.001$, $DF = 562$), and the functional coupling during the 0-back task is not significantly correlated with performance (see panel *(B)*; repeated measures correlation; $r = -0.0195$, $p = 0.643$, $DF = 562$). Similarly, during the 2-back task the frontoparietal activity is correlated with performance (see panel *(C)*; Pearson correlation; $r = 0.115$, $p = 0.0047$), while the default mode activity is not significantly correlated with performance (see panel *(D)*; Pearson correlation; $r = 0.059$, $p = 0.15$).

Supplementary Note 4 : Alternate parcellation

In the main text, we chose to divide the brain into 400 discrete non-overlapping regions where each region is assigned to a functional system. However, recent studies commonly report results across at least two parcellations, as it remains unclear whether a single parcellation is optimal for all studies and the testing of all hypotheses. While there have been several attempts to empirically define the optimal number of brain areas [30, 31], the brain is organized hierarchically [32], and so the number and size of parcellations to use becomes dependent on the question to be addressed. One of the requirements we imposed on our choice of parcellation was the ability to discern inhomogeneity in the frontoparietal system, and so we required subdivisions within that system. To reduce parcellation complexity, we also wanted a small number of total brain parcels. The 400 region parcellation provided us with a relatively small total number of parcels, while maintaining a relatively high resolution within the frontoparietal system (61 parcels). However, we were interested to see how robust our results were with respect to the choice of parcellation, and thus we considered a second, lower resolution parcellation composed of 100 regions [33]. Because of the difference in resolution, we expected that system-level results would be consistent across parcellations, but that subnetwork results might be altered.

In line with our hypothesis, we found that the system-level results were unaltered (Supplementary Figure 4), while the subnetwork level results did change modestly as a function of parcellation. The frontoparietal system can still be separated into two distinct subnetworks where one displays activity that is correlated with the activity of the default mode system (subnetwork (B)) and the other displays activity that is anticorrelated with the activity of the default mode system (subnetwork (A)) (Supplementary Figure 5A, B). Furthermore, as before, increasing subnetwork (A) activity is related to decoupling of the frontoparietal and default mode systems (Supplementary Figure 5C), and increasing subnetwork (B) activity is related to coupling of the frontoparietal and default mode systems (Supplementary Figure 5D). The primary difference between results obtained from the 400 region parcellation and from the 100 region parcellation is the anatomical distribution of the subnetworks over the cortical surface. Whereas before we observed subnetworks that were partially constrained to different hemispheres, we now observe sub-networks constrained to either the medial (subnetwork (B)) or lateral (subnetwork (A)) surfaces. These findings suggest that the large-scale architecture of

the 100 region parcellation is more sensitive to differences in dynamics that are maintained across both hemispheres, while the smaller-scale architecture of the 400 region parcellation is more sensitive to differences in dynamics that vary across the two hemispheres. More generally, it is well known that including a smaller number of regions, where regions are larger on average, enables the examination of larger hierarchical dynamics [33], resulting in the identification of different subnetworks.

It is important to emphasize that this division of the frontoparietal system is no less valid than the one presented in the main text, but rather is reflective of dynamics on a larger physical scale. Indeed, it has been reported that the medial parietal cortex activates during the observance of social interactions [34], which requires recruitment of the default mode system [35]. The medial parietal cortex is part of our subnetwork (B), which is heavily integrated with the default mode system and drives functional coupling between the frontoparietal and default mode systems. Furthermore our subnetwork (A) is largely composed of the prefrontal cortex, a region long established as critical to working memory [36, 37, 38], and drives the anticorrelations between the frontoparietal and default mode systems, which in turn could provide the necessary functional buffer from internal trains of thought.

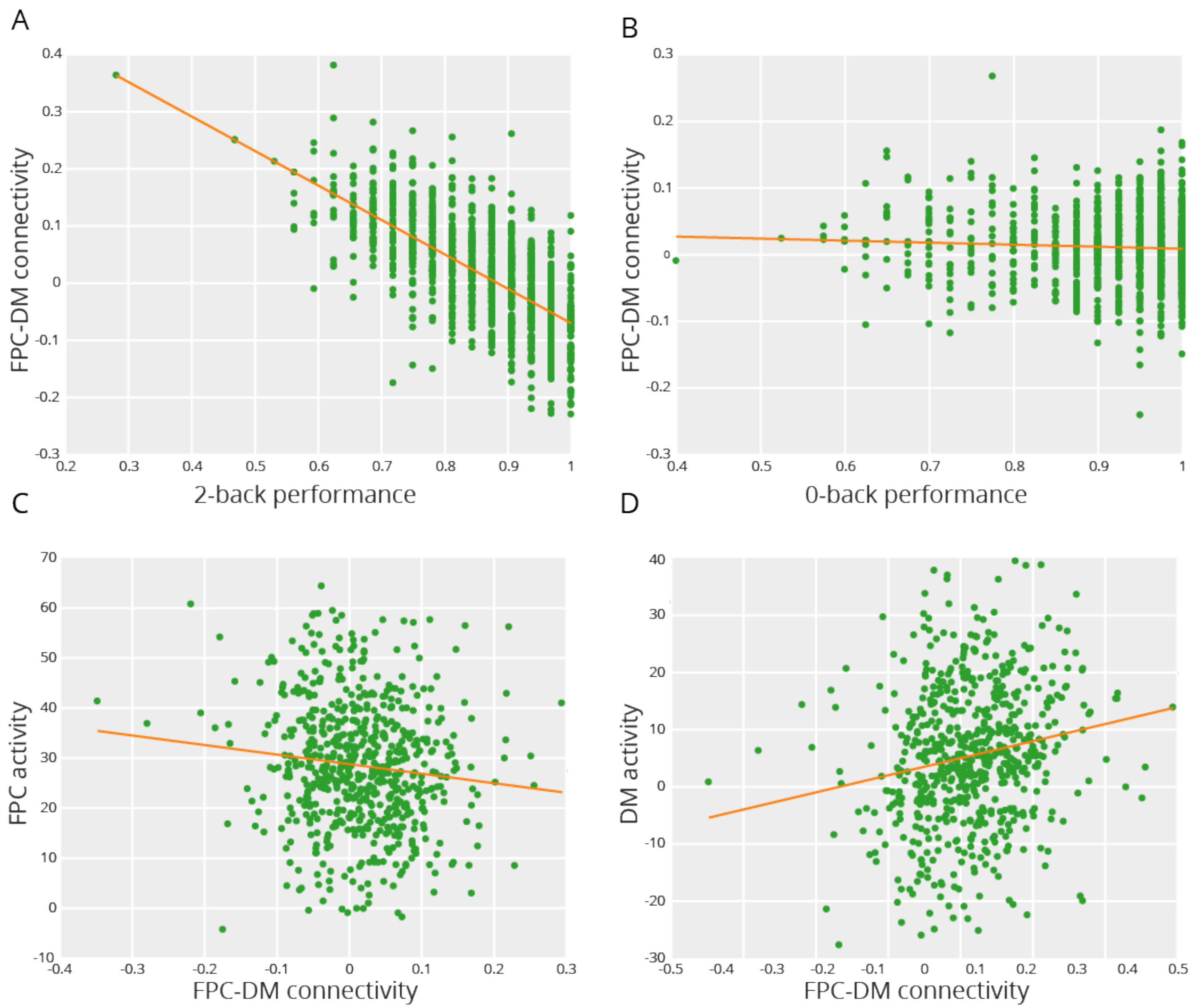

Supplementary Figure 4: **Replication of results with a different parcellation.** Using the alternate parcellation, the relationships between connectivity and behavior remained unchanged. Specifically, the functional coupling between the frontoparietal and default mode systems during the 2-back working memory task was negatively correlated with performance (see panel *(A)*; repeated measures correlation; $r = -0.4498$, $p < 0.001$, $DF = 558$), and the functional coupling between the two systems during the 0-back task was not significantly correlated with performance (see panel *(B)*; repeated measures correlation; $r = -0.0347$, $p = 0.409$, $DF = 562$). Similarly, during the 2-back task the frontoparietal activity was negatively correlated with performance (see panel *(C)*; Pearson correlation; $r = -0.1121$, $p = 0.0047$), while the default mode activity was significantly positively correlated with performance (see panel *(D)*; Pearson correlation; $r = -0.1826$, $p = 3.55 \times 10^{-6}$).

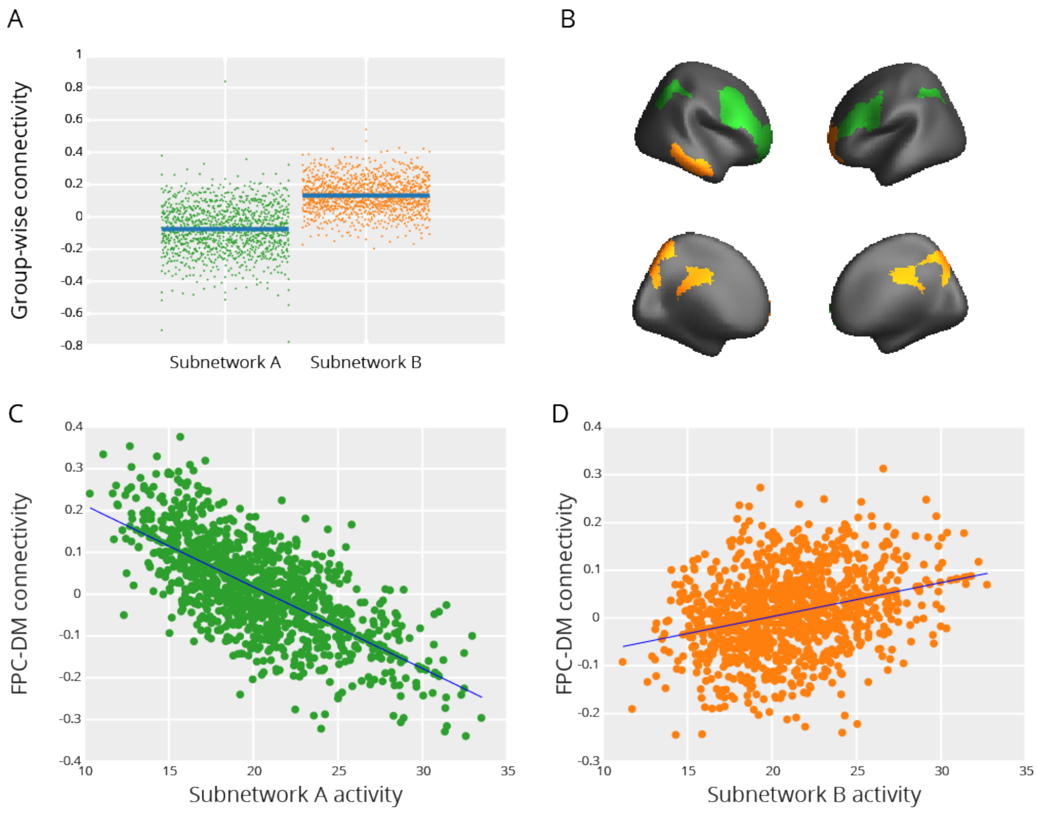

Supplementary Figure 5: **Replication of subnetwork results with a different parcellation.** *(A)* Using the alternate parcellation, we found that two frontoparietal subnetworks exist with different relationships to the default mode system. Specifically, we found that subnetwork (A) is negatively functionally connected with the activity of the default mode system (mean $r = -0.076$, $p < 0.001$, $t(1206) = -18.1$, 95% CI: (-0.084,-0.068)), while subnetwork (B) is positively functionally connected with the default mode system (mean $r = 0.132$, $p < 0.001$, $t(1206) = 41.6$, 95% CI: (0.126, 0.138)). The two groups significantly differ from each other according to both our multilevel model ($\beta = -0.20892$, $p < 0.0001$, $t(1769) = -41.3$, $SE = 0.0050548$, $n = 2414$). *(B)* The two groups projected onto the cortical surface. *(C)* Subnetwork (A) activity is negatively correlated with the functional coupling between the frontoparietal and default mode systems ($\beta = -0.0088$, $p < 0.001$, $t(1204) = -17.2$, 95% CI: (-0.00989, -0.00787)). *(D)* Subnetwork (B) activity is positively correlated with the functional coupling between the frontoparietal and default mode systems ($\beta = 0.00435$, $p < 0.001$, $t(1204) = 6.2$, 95% CI: (0.00298, 0.00571)) when using both as independent variables in a robust linear mixed effects model.

Supplementary Note 5 : Alternative functional connectivity measure

In the main text, we computed functional connectivity using the Pearson correlation coefficient. However, there are numerous methods used to establish functional connectivity between time series in neuroimaging [39], with correlation-based measures accounting for just a single subset of these methods. In order to demonstrate the robustness of our results, we sought to reproduce our main findings using a measure of functional connectivity unrelated to the correlation. We chose to use wavelet coherence due to its established usage in neuroimaging [40, 41, 19, 42], and we chose wavelet scale 2, which corresponds to the relevant frequency range of 0.06 Hz - 0.12 Hz [41]. Rather than probing a single linear relationship between time series, coherence is a measure of the cross-correlation between time series [39]. Similar to the Pearson correlation coefficient, the wavelet coherence is undirected. However, unlike the Pearson correlation coefficient, wavelet coherence is bounded in [0,1], and thus anti-correlation cannot be observed. Importantly, our main findings hold using this different estimate of functional connectivity (Supplementary Figure 6).

Specifically, we found that the strength of the functional connection between the default mode and frontoparietal systems remains related to behavioral performance on the 2-back working memory task ($\beta = 0.11817$, $p < 0.0001$, $t(555) = 6.2466$, $SE = 0.018844$, $n = 1192$; Supplementary Figure 6A), and that the strength of the intersystem connection remains correlated with frontoparietal activity ($\beta = 28.759$, $p = 0.0002$, $t(560) = 3.7279$, $SE = 7.7145$, $n = 1203$; Supplementary Figure 6B). The sign of the relationship between the functional connection strength and behavioral performance is inverted relative to the Pearson correlation results, due to the fact that coherence is always positive. Community detection to identify two distinct subnetworks of the frontoparietal system resulted in a slightly different split than that reported in the main manuscript (Supplementary Figure 6C), although the two share some general characteristics. Importantly, the coherence-derived subnetworks are functionally distinct, with subnetwork (A) less strongly connected to the default mode than subnetwork (B). Furthermore, in keeping with our main results, (i) the activity of the subnetwork more weakly connected to default mode (subnetwork (A)) displays activity that is negatively correlated with the functional coupling between the frontoparietal and default mode systems (Supplementary Figure 6E), and (ii) the activity of the subnetwork more strongly connected to the default mode system (subnetwork (B)) displays activity that is positively correlated with the functional coupling

between the default mode and frontoparietal systems (Supplementary Figure 6F).

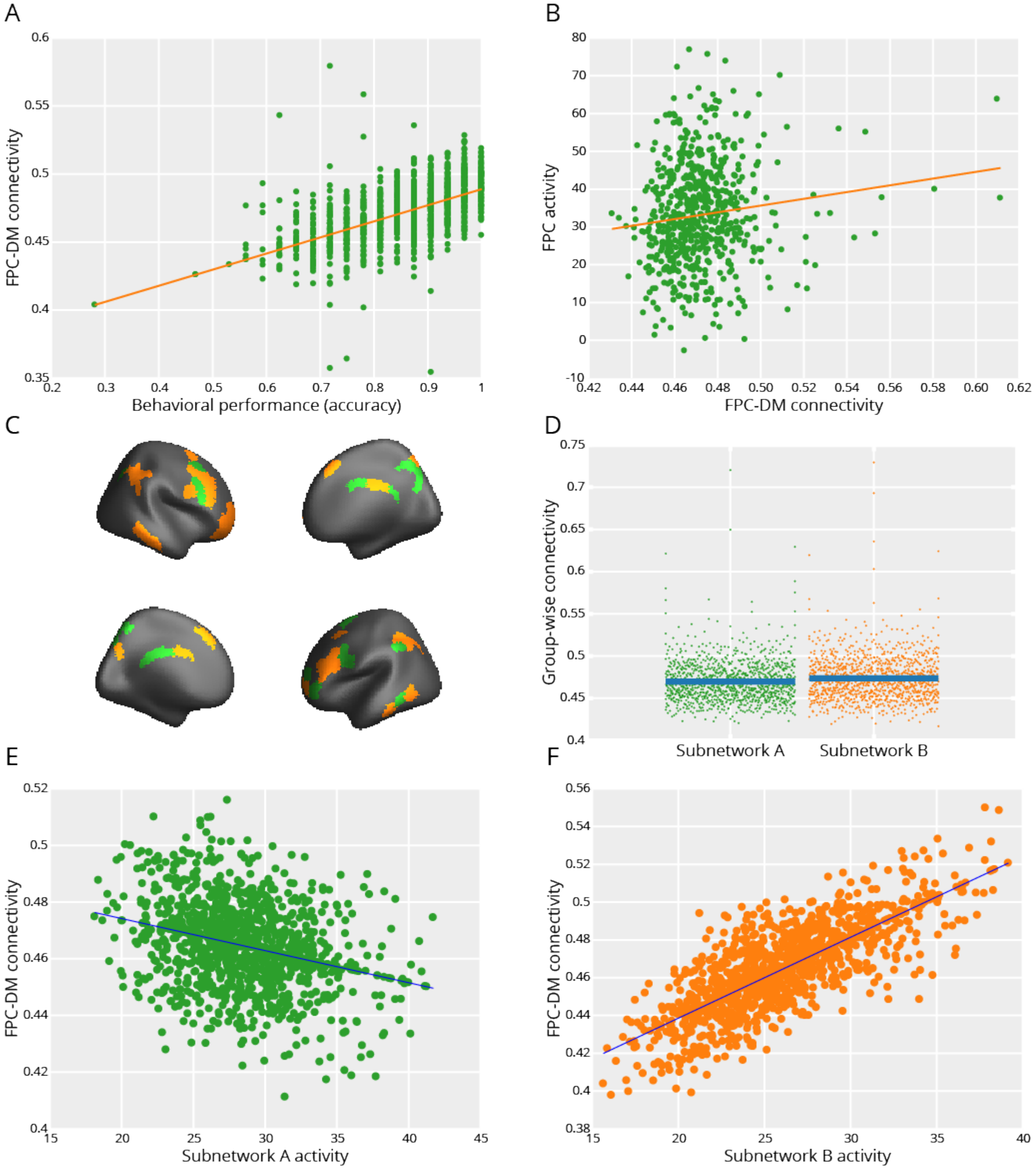

Supplementary Figure 6: **Replication of results with a coherence-based estimate of functional connectivity.** *(A)* Behavioral performance on the 2-back working memory task relates to the functional coupling between the frontoparietal and default mode systems (repeated measures correlation; $r = 0.356$, $p < 0.0001$, $DF = 555$). *(B)* Frontoparietal activity relates to the functional coupling between the frontoparietal and default mode systems (Pearson correlation; $r = 0.1222$, $p = 0.0020$). *(C)* Community detection via the weighted stochastic block model reveals a 2 subnetwork fractioning of the frontoparietal system. *(D)* Subnetwork (A) is less connected to the default mode system (mean $= 0.469$, $p < 0.001$, $t(1174) = 705$, 95% CI: (0.468, 0.471)) than subnetwork (B) (mean $= 0.473$, $p < 0.001$, $t(1174) = 793$, 90% CI: (0.472, 0,475)). The two groups significantly differ from each other according to a multilevel model ($\beta = 0.0038179$, $p < 0.0001$, $t(1769) = 4.2$, $SE = 0.00089474$, $n = 2414$). Fitting a single robust model accounting for the activity of both subnetworks demonstrates *(E)* that subnetwork (A) activity is negatively correlated with the functional coupling between the frontoparietal and default mode systems ($\beta = -0.003055$, $p = 0.0095$, $t(1199) = -2.35$, 95% CI: (-0.00056, -0.000005)), while *(F)* subnetwork (B) activity is positively correlated with the functional coupling between the frontoparietal and default mode systems ($\beta = 0.00148$, $p < 0.001$, $t(1199) = 12.1$, 95% CI: (0.001246, 0.000172)).

Supplementary Note 6 : Accounting for the effect of spatial distance on gene coexpression between brain regions

Gene coexpression is known to decrease as a function of distance between the two regions being studied [43]. Thus, any observed difference in gene coexpression between two subnetworks could be due rather trivially to differences in distance spanned by the regions in the two subnetworks. To ensure that distance was not driving our observations, we assessed the pairwise distances between regions in subnetwork (A), the pairwise distances between regions in subnetwork (B), and the pairwise distances between all regions in the frontoparietal system, which is comprised of regions in both subnetwork (A) and subnetwork (B). We defined the pairwise distance between two parcels to be the Euclidian distance between the mean X, Y, and Z coordinates of the respective parcels in volumetric space. Specifically, we drew 50000 bootstrap samples of regions in subnetwork (A), subnetwork (B), and the frontoparietal system as a whole. We then calculated (i) the difference between the mean subnetwork (A) distance and the mean subnetwork (B) distance, (ii) the difference between the mean subnetwork (A) distance and the mean frontoparietal system distance, and (iii) the difference between the mean subnetwork (B) distance and the mean frontoparietal system distance. To conclude the analysis, we determined whether the 95% confidence interval for any of these difference distributions included zero.

After performing these computations, we found that the difference in mean within-subnetwork distance between subnetwork (A) and subnetwork (B), as well as the difference in mean within-subnetwork distance between subnetwork(A) (or (B)) and the mean frontoparietal network distance, were all not significantly different than 0. Specifically, we found that the difference in mean within-subnetwork distance between subnetwork (A) and subnetwork (B) is not significantly different than 0 (95% CI : $[-8.5979, 3.7972]$), the difference in mean within-subnetwork distance between subnetwork (A) and mean within frontoparietal network distance is not significantly different than 0 (95% CI : $[-6.6320, 2.5436]$), and the difference in mean within-subnetwork distance between subnetwork (B) and the mean frontoparietal network distance is not significantly different than 0 (95% CI : $[-9.6414, 0.8261]$). Given these findings, inter-regional distance is unlikely to explain the observed differences in gene coexpression in the two subnetworks.

Supplementary Note 7 : Subnetwork genetic differences

The main text noted significant genetic differences between subnetworks, whereas here in the supplement we address the ways in which the subnetworks were genetically different. Specifically, for each subnetwork, we calculated the mean expression for each of the 16699 genes. We then performed a genetic ontology enrichment analysis using GOrilla [44]. Briefly, this analysis sorts each gene into pre-defined bins, called ontologies, and performs a statistical test to determine whether expression in that ontology is greater than expected. This analysis provided us with two lists of enriched ontologies, one for each subnetwork. In order to better organize these lists, we wished to group related ontologies with each other. We therefore employed REvIGO [45], a tool that determines the semantic similarity between each ontology's 'process' annotation. This step allowed us to create a semantic network for each subnetwork's enrichment list, where nodes were enriched ontologies and edges were the degree of semantic similarity between annotations. Lastly, to investigate the extent to which each subnetwork expressed groups of semantically related ontologies, we estimated the community structure of the two networks by employing a modularity maximization algorithm. The two networks, along with their nodal community assignments, can be seen in Figs. Supplementary Figure 7 and Supplementary Figure 8, respectively. We investigated the structure of these semantic networks to probe how they might relate to the different functions of the frontoparietal subnetworks.

It is immediately evident that, although the two semantic networks have similar numbers of nodes (70 in subnetwork (A); 77 in subnetwork (B)), the edge density of these two networks is quite different. The sum of edge weights in the semantic network of subnetwork (B) is roughly twice that observed in the semantic ontology network of subnetwork (A). Intuitively, this distinction indicates that the enriched genetic ontologies of subnetwork (B) are semantically more similar than the enriched genetic ontologies of subnetwork (A). At first glance, this observation may appear counterintuitive; subnetwork (B) is more spatially distributed over the cortex than subnetwork (A), and one might speculate that the spatial diversity would be accompanied by a corresponding genetic diversity. However, such a speculation is not supported by the data. Instead, our observations are consistent with the functional roles of the two subnetworks; subnetwork (A) is closely related to the default mode system which supports a range of cognitive processes, and may require an equally diverse range of genetic

processes to support that function.

Several coarse-grained similarities exist between the ontologies of the two subnetworks. Subnetwork (A) community 1 and subnetwork (B) community 7 contain genes involved in stimulus detection. Subnetwork (A) community 3 and subnetwork (B) community 6 contain genes involved in cellular component and cellular projection organization. Subnetwork (A) communities 5 & 8 and subnetwork (B) community 5 contain genes involved in development and differentiation. Subnetwork (A) community 6 and subnetwork (B) community 2 contain genes involved in hormonal regulation. Subnetwork (A) community 7 and subnetwork (B) community 1 contain genes involved in ion transport. Communities also exist that are not represented in both subnetworks. Subnetwork (A) community 4 contains genes involved in cellular movement, and community 2 contains genes involved in transmembrane transport. Subnetwork (B) community 3 contains genes involved in behaviors, while community 8 contains genes involved in cellular signaling pathways, and community 4 contains genes involved in various multicellular organism processes.

Although an in-depth discussion interpreting the above findings is outside the scope of this work, we will note a few broad features that may be relevant to the observed functional differences of the subnetworks. First, we note that subnetwork (A) contains 4 enriched genetic ontologies relating to stimulus detection, where subnetwork (B) contains 9 enriched ontologies. This difference may suggest that cells contained within subnetwork (B) may be more tuned to the detection of stimuli relative to the cells contained within subnetwork (A). Broadly consistent with this interpretation, subnetwork (B) is functionally aligned with the dorsal attention network, which plays a role in directing attention to external stimuli [5], a function that may be reflected in the underlying genetic expression of these cells. Second, we can observe that the semantic network for subnetwork (B) contains a community for external behavior, while the semantic network for subnetwork (A) does not. In other words, the cells contained within subnetwork (B) are enriched for ontologies relating to behavior, while the cells contained in subnetwork (B) display no such enrichment. This finding is also consistent with our interpretation of subnetwork (B)'s alignment with the dorsal attention system, as supporting the completion of tasks involving external stimuli, certainly relevant to the observed enrichments for locomotion and eating.

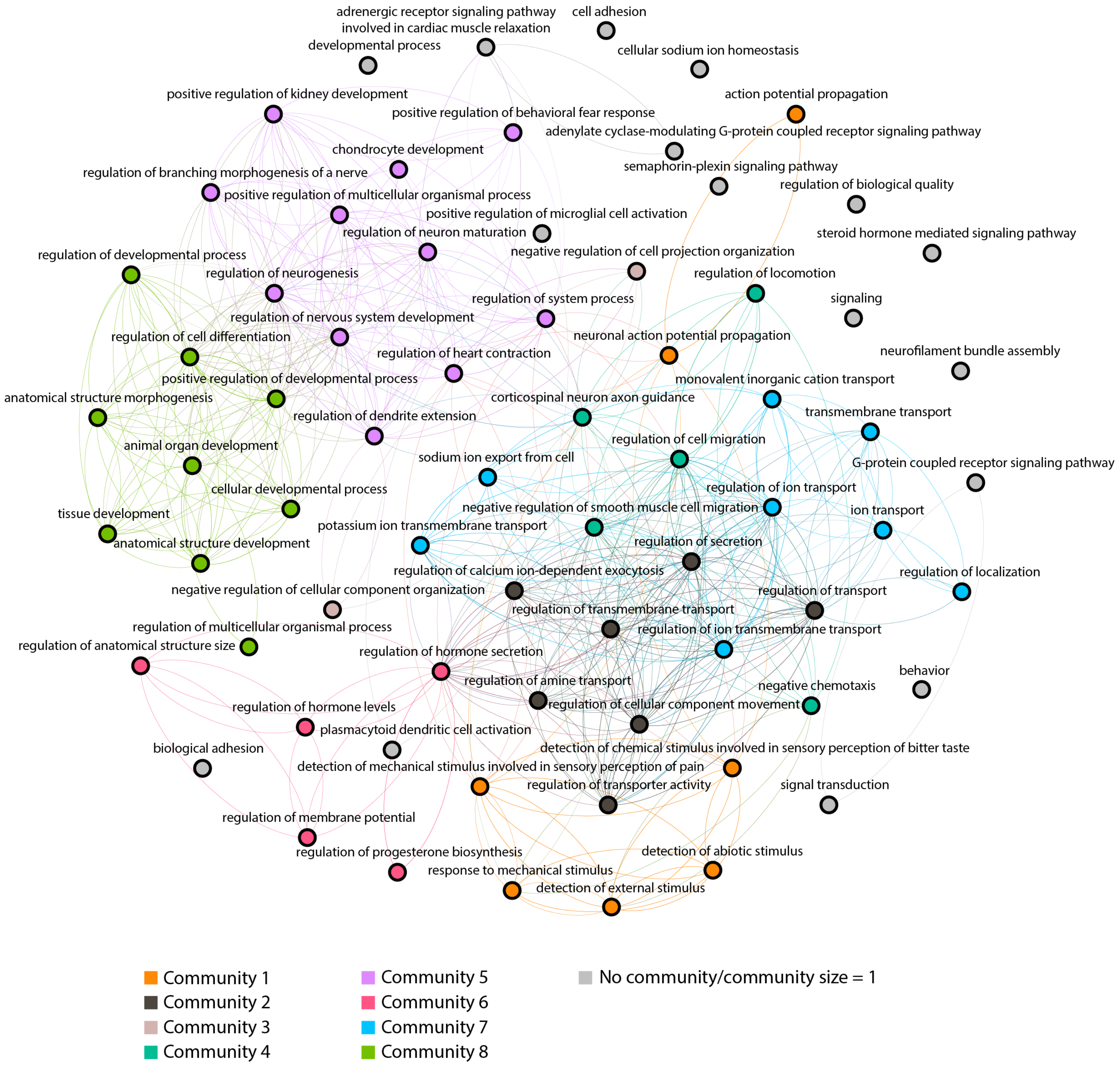

Supplementary Figure 7: **Genetic ontology semantic network for Subnetwork (A).**

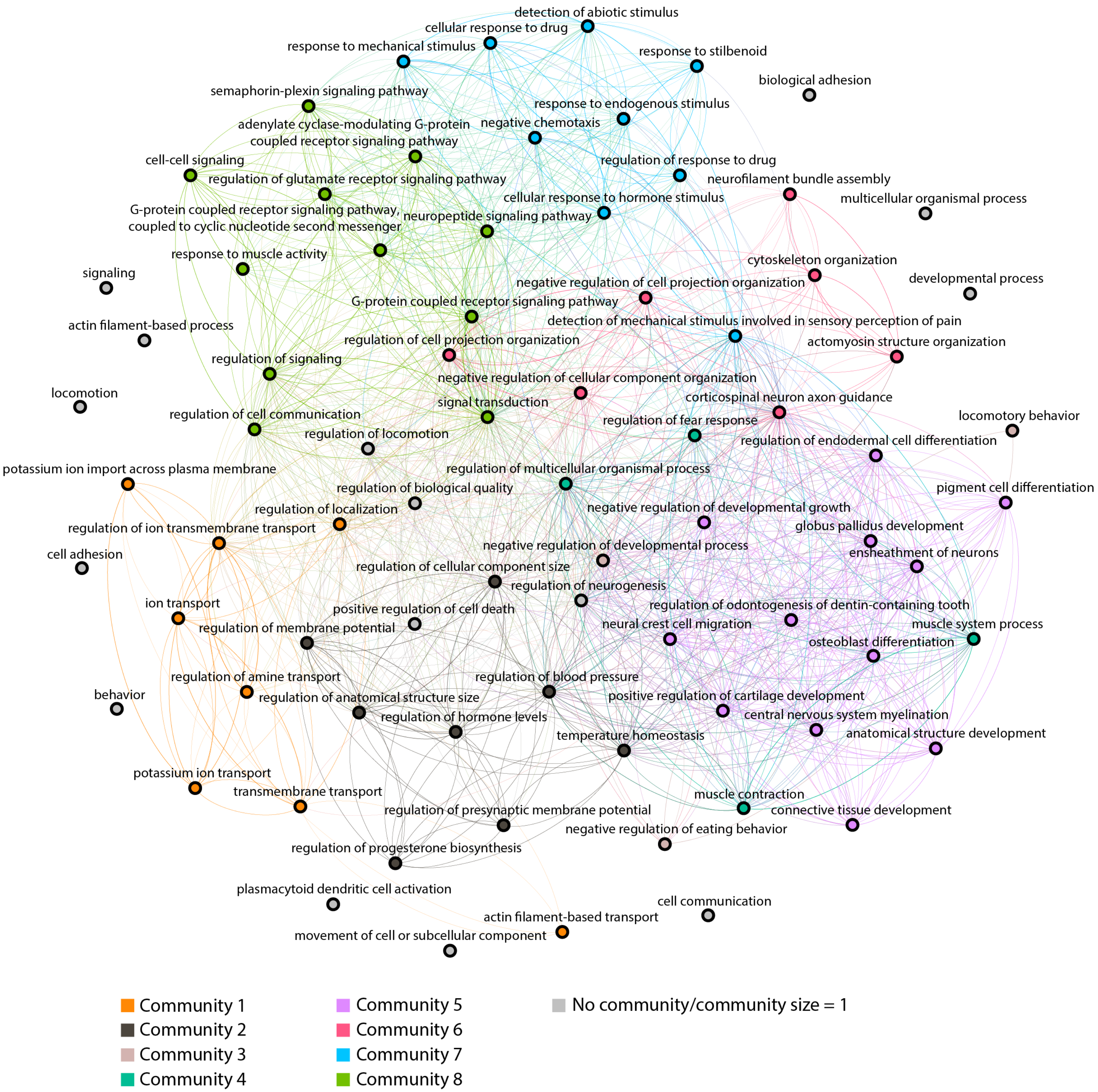

Supplementary Figure 8: **Genetic ontology semantic network for Subnetwork (B).**

Supplementary Note 8 - Accounting for indirect structural connections

In addition to the direct structural connections considered in the main text, indirect structural connections have been shown to contribute meaningfully to functional connectivity, although to a smaller degree than direct structural connections [46]. Furthermore, the shortest structural path between two regions may be an indirect rather than a direct path [47]. Taking these prior observations into account, we sought to understand how subnetwork structural connections would be affected by accounting for indirect connections. We therefore examined connections that (i) were direct or traversed a single intervening region, (ii) were direct or traversed up to two intervening regions, or (iii) all white matter connections regardless of the number of intervening regions. When considering up to one intervening region (up to path length of 2, summing path strengths), subnetwork (B) is more strongly connected to the dorsal attention system than subnetwork (A) ($\beta = 0.000509$, $p < 0.0001$, after Bonferroni correction for multiple comparisons $t(2412) = 98.3$), and subnetwork (A) is more strongly connected to the default mode system than subnetwork (B) ($\beta = -0.0014$, $p < 0.0001$ after Bonferroni correction for multiple comparisons, $t(2412) = -169$). When considering all possible path lengths (summing over all path lengths), subnetwork (B) is more strongly connected to the dorsal attention system than subnetwork (A) ($\beta = 0.0000312$, $p < 0.0001$ after Bonferroni correction for multiple comparisons, $t(2412) = 100$), and subnetwork (A) is more strongly connected to the default mode system than subnetwork (B) ($\beta = -0.000089$, $p < 0.0001$ after Bonferroni correction for multiple comparisons, $t(2412) = -162$). Further, across subjects we found that the less structurally connected the two subnetworks were, the better individuals tended to perform on the 2-back working memory task ($r = -0.0766$, $p = 0.03$).

Supplementary Note 9 - Estimation of GLM functional activation

Functional activations and contrasts for each subject are included in the publicly available HCP S900 release. Complete details have been documented elsewhere [48], and therefore we provide only a brief summary. In the GLM analysis of the functional imaging data collected during the working memory task, 8 model predictors were included: 0-back body, 0-back face, 0-back place, 0-back tool, 2-back body, 2-back face, 2-back place, and 2-back tool. Activity estimates were calculated for each of these conditions by using FEAT in FSL to produce linear contrasts against baseline fixation, as well

as between conditions. In order to move these results to parcel-space, we simply averaged activity within each parcel to obtain one activity value per parcel.

Supplementary Note 10 : Effect of biologically motivated parameters on model results

Our model has two biologically-motivated parameters: (1) the coupling strengths between oscillators, which are derived from the empirical anatomical connectivity matrix, and (2) the frequencies of oscillators, which are estimated from resting-state data. In addition, to investigate the empirical result that subnetwork A and B activity modulate the FP-DM functional coupling in particular ways, we also incorporated the effect of differential activations of subnetwork A and subnetwork B activity into the model by changing the bifurcation parameter of nodes comprising the two subnetworks. The value of the bifurcation parameter is of course not the biophysical mechanism underlying the activation level of subnetwork A or B. However, tuning this parameter still allowed us to study the effect of specific variations in the activity of subnetwork A or subnetwork B on FP-DM functional connection strengths.

It is important to discuss and comment on how the two main biophysically-motivated parameters – the structural coupling strengths and the natural frequencies – may provide the basis for the observed modulation of the FP-DM coupling. Indeed, a large body of computational modeling work supports the hypothesis that both anatomical coupling and dynamical time-scales influence large-scale patterns of brain activity such as those observed in fMRI [49, 50, 51, 52, 53]. Importantly, structural connectivity provides the scaffold upon which large-scale neural activity travels, and dictates which brain areas can directly influence which other areas [54]. It is now well-known that, regardless of other details, functional interactions between distributed parts of the brain can often be tightly related to network structure [46]. Given these findings, it is then also reasonable to expect that anatomical connectivity should at least partially determine how an increase or decrease in activity in a subset of regions will propagate throughout the network and affect activity levels and functional interactions in and between downstream areas. In particular, past work has highlighted the role of structural connectivity in guiding the widespread effects of local perturbations of neural activity [55, 56], and has also uncovered links between brain network structure and the ability of certain areas to control brain network dynamics [57, 9]. It is therefore reasonable to assume that structural coupling architecture should play a role in how FP-DM functional connection strengths manifest and are modulated by changes in neural activity in certain parts of the system. Furthermore, we also know from fundamental

theory and simplified models that the natural frequencies of units in a networked-system influence how easily they can synchronize with one another [58, 59]. Therefore, the strengths and locations of functional interactions in brain networks – and how those interactions are tuned when activity levels are perturbed – should depend on how intrinsic frequencies are distributed among network components [60, 61, 62, 63]. Indeed, large-scale brain network models have shown that perturbations to intrinsic frequencies can lead to widespread reconfigurations of functional interactions [64, 65, 60], demonstrating the importance of considering the distribution of natural frequencies in governing brain network dynamics.

It is also important to note a few additional points. First, while we are able to find locations in the global coupling strength (G) and bifurcation parameter (a) phase-space where the biologically-informed version of the model can recover the empirical findings, it does not recover the results for all combinations of those two parameters. Thus, structural connectivity and natural frequencies are not the only features of the system that matter. The overall coupling strength and the baseline value of the bifurcation parameter in the model also seem to be important. While we believe it is beyond the scope of the current study, it would be interesting in future work to more carefully examine the role or relative importance of other factors. Second, it would be interesting to understand if incorporating more task-specific or subject-specific aspects of brain activity into the model could improve its performance or allow us to further understand individual variability. For example, natural frequencies, global couplings, and bifurcation parameters could be informed from person-specific resting or task data, and chosen, for example, such that simulated dynamics most closely match results from individual human scans. Finally, it is important to state that the results from the model certainly do not rule out other possible mechanisms or factors driving the empirical results, but rather provide intuition about some variables that may be important and that may play a role.

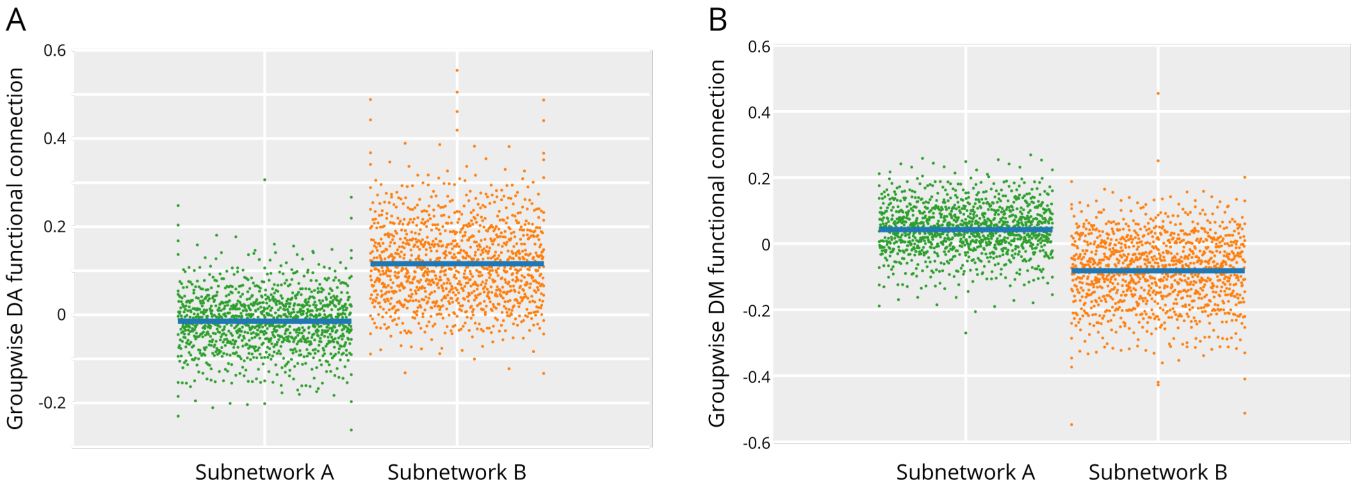

Supplementary Figure 9: **Frontoparietal subnetwork connectivity.** *(A)* Subnetwork (A) is negatively functionally connected to the dorsal attention system (mean $r = -0.014$, $p < 0.001$, t(1206) = -7.22, 95%CI: (-0.18, -0.11)), while subnetwork (B) is positively functionally connected to the dorsal attention system (mean $r = 0.12$, $p < 0.001$, $t(1206) = 39$, 95%CI: (0.11, 0.12)). Using a multilevel model, we found that these correlations are significantly different ($\beta = 0.13057$, $p < 0.0001$, $t(1769) = 46.8$, $SE = 0.0027885$, $n = 2414$). *(B)* Subnetwork (A) is positively functionally connected to the default mode system (mean $r = 0.042$, $p < 0.001$, $t(1206) = 20$, 95% CI: [0.038, 0.046]), while subnetwork (B) is negatively functionally connected to the default mode system (mean $r = -0.082$, $p < 0.001$, $t(1206) = -27$, 95%CI: (-0.088, -0.076)). Using a multilevel model, we found that these correlations are significantly different ($\beta = -0.12469$, $p < 0.0001$, $t(1769) = -37.4$, $SE = 0.0033272$, $n = 2414$). Note that for visualization purposes, the data was not visually adjusted to account for subject repeated measures effects, whereas all reported statistics do take this into account.

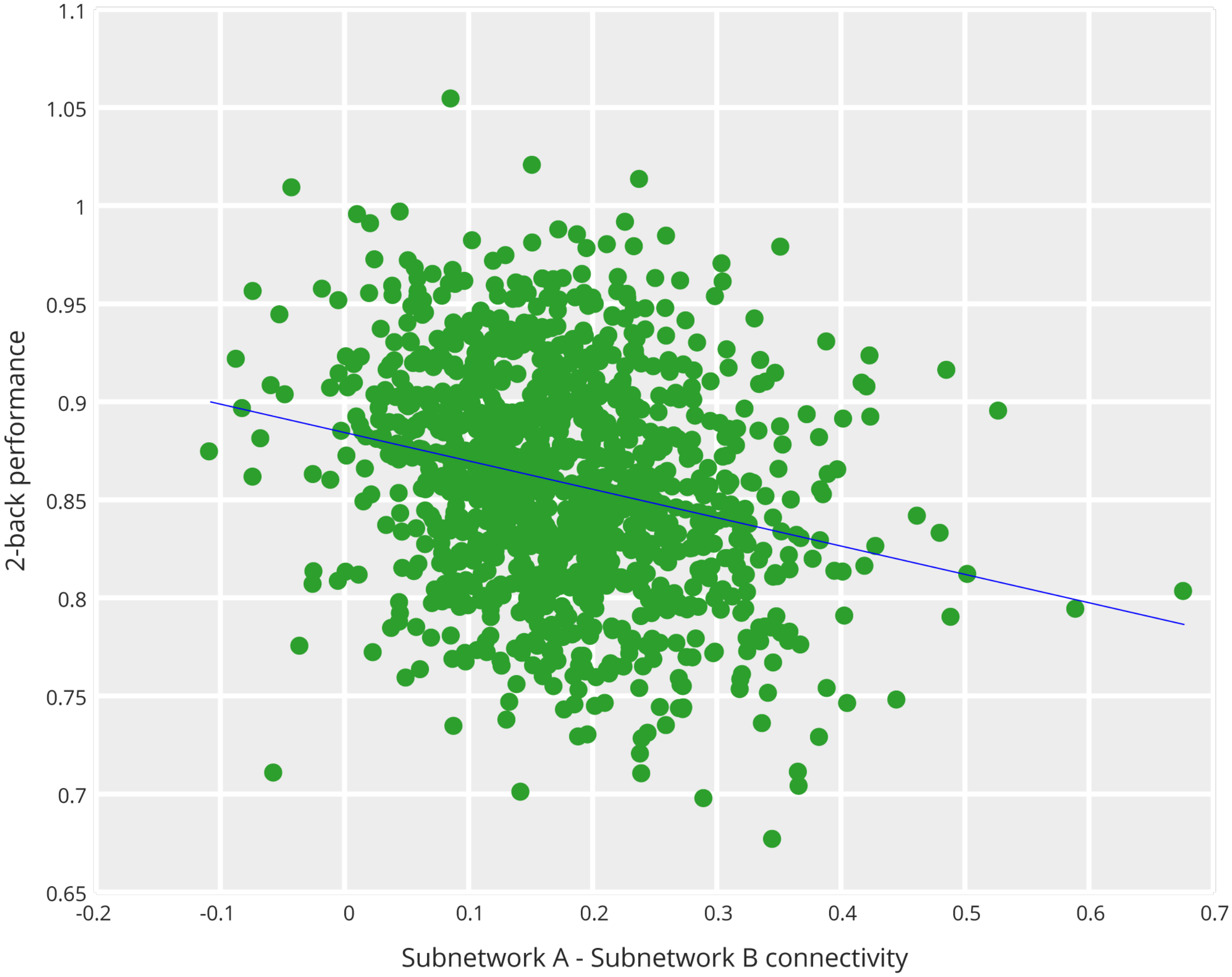

Supplementary Figure 10: **Frontoparietal subnetwork connectivity is related to performance.** As the two frontoparietal subnetworks display more correlated time series, performance on the 2-back working memory task worsens (Pearson correlation coefficient $r = -0.8544$, $p = 0.0003$).

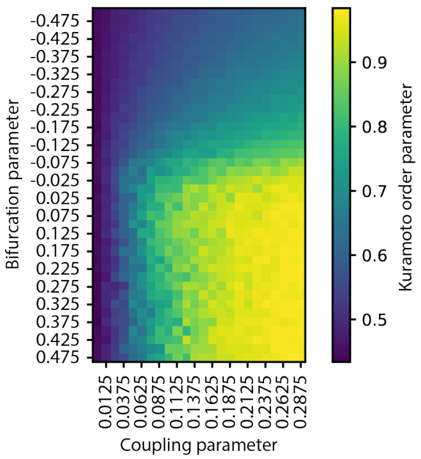

Supplementary Figure 11: **Determination of Hopf bifurcation model parameters.** Kuramoto order parameter as a function of the coupling parameter and of the bifurcation parameter for the 4 oscillator model.

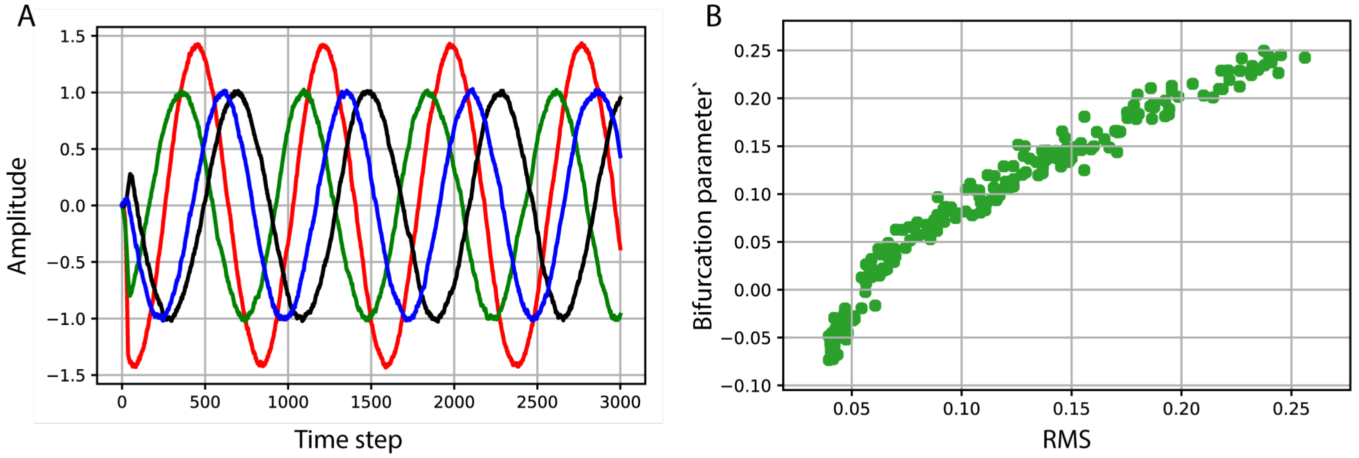

Supplementary Figure 12: **Relationship between signal amplitude and bifurcation parameter from the computational model of brain system dynamics.** *(A)* Here we show a simulation of a 4 node network. The dynamics of each unit are described by the normal form of a Hopf bifurcation (see Methods in the main text), and exhibit oscillatory activity at the chosen bifurcation parameters. The green, blue, and black oscillators have identical bifurcation parameters equal to 1. The red oscillator has a bifurcation parameter equal to 2, which serves to increase the amplitude without changing other features of the signal. *(B)* Here we simulate a 4 node network while varying the bifurcation parameter of one node, and measuring the root mean square (RMS) of the resulting signal of the same node to show that the manipulation of the bifurcation parameter linearly alters the RMS of the resulting time series (Pearson correlation coefficient $r = 0.9644$, $p < 0.0001$).

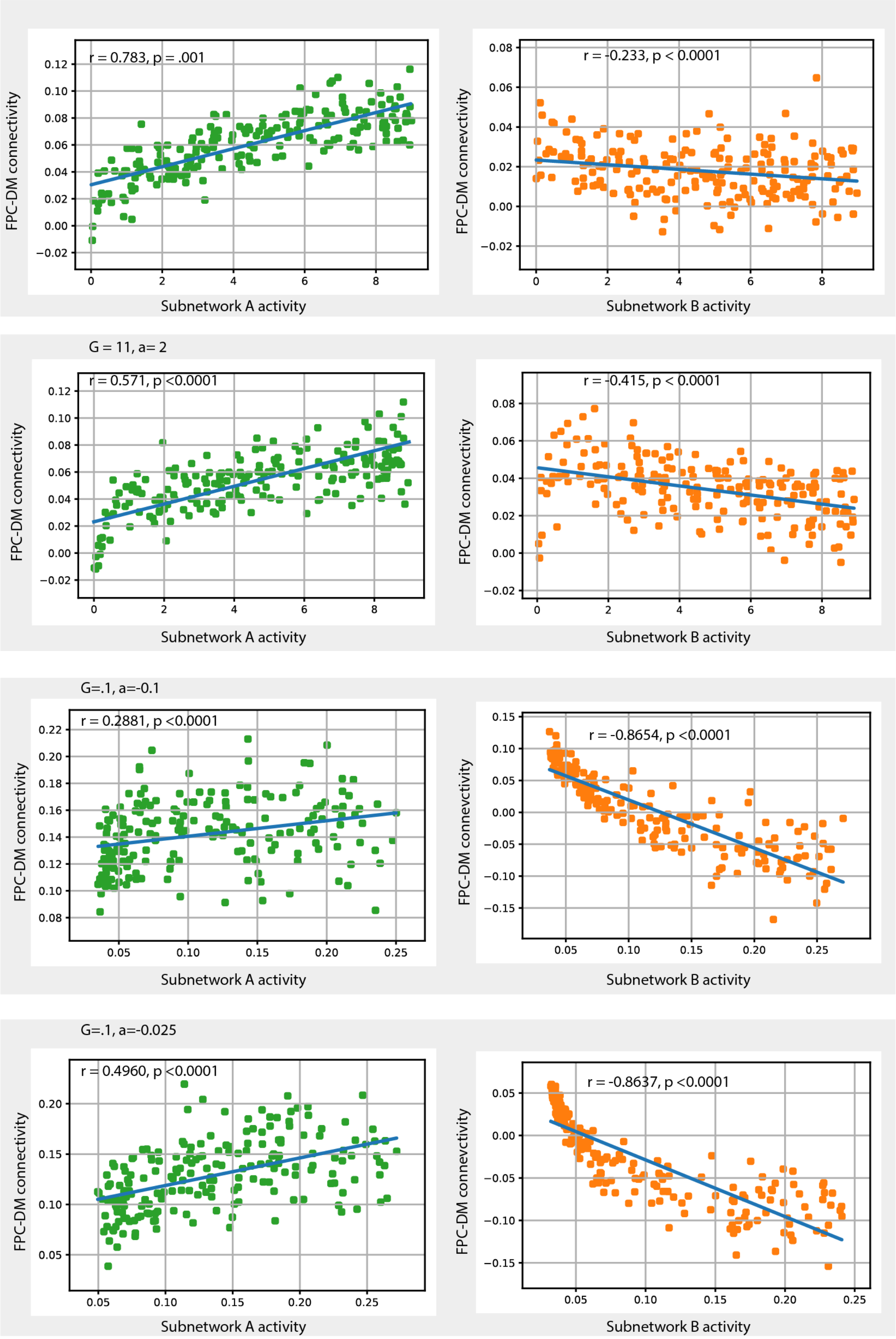

Supplementary Figure 13: **Results from the dynamical model at different parameter values.** These figures reproduce the results of 3 for the 4 oscillator model in the main manuscript at different values of the coupling and bifurcation parameters for the 3 nodes where activity is not being modulated.

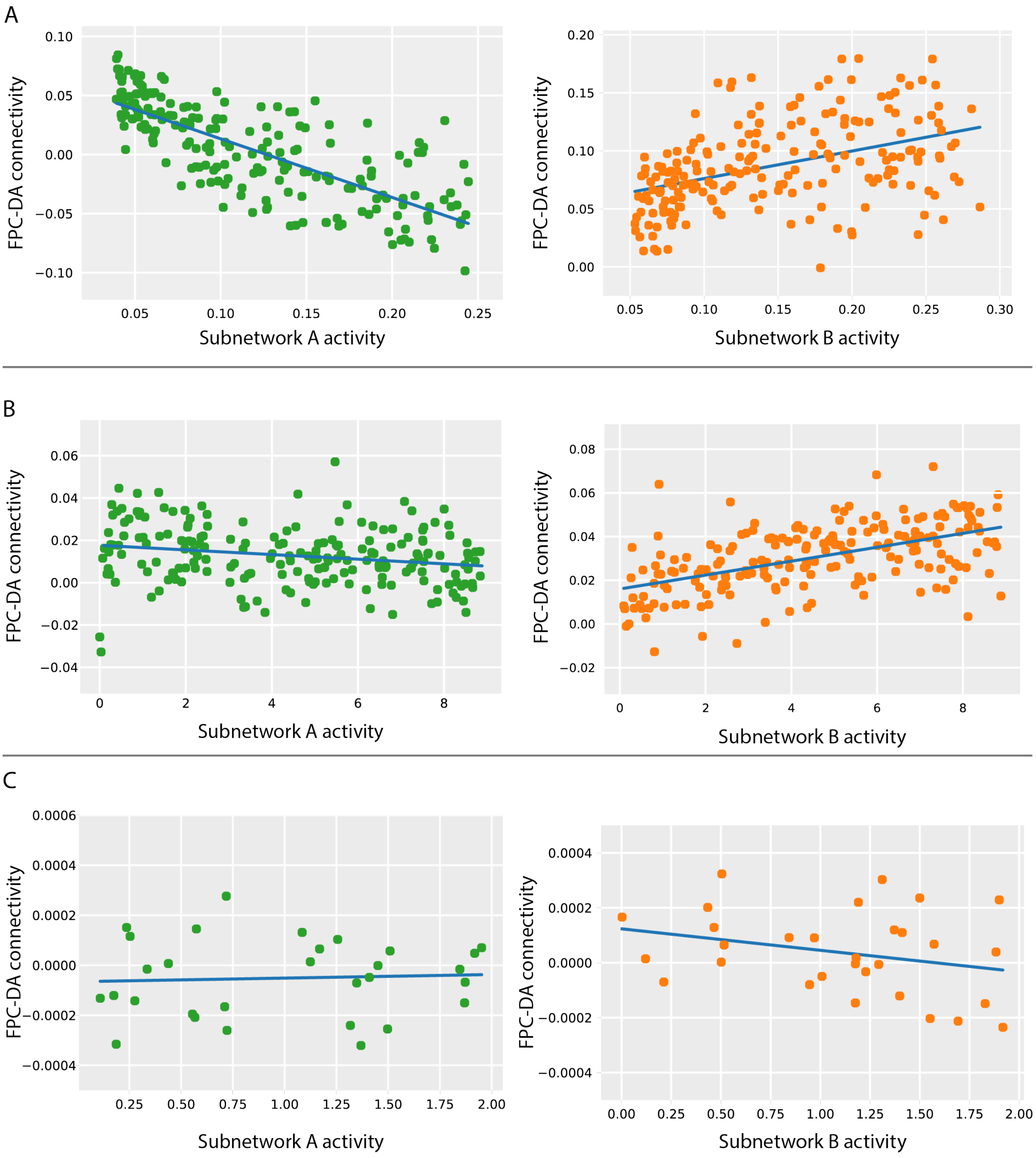

Supplementary Figure 14: **Simulated frontoparietal and dorsal attention dynamics.** *(A)* In our 4 oscillator model, increasing the amplitude of subnetwork (A) (right) caused a decrease in the functional coupling between the frontoparietal and dorsal attention systems (Pearson correlation coefficient $r = -0.216$, $p = 0.001$), and increasing the amplitude of subnetwork (B) (left) in the computational model caused an increase in functional coupling between the frontoparietal and dorsal attention systems (Pearson correlation coefficient $r = 0.538$, $p < 0.0001$). *(B)* The same experiment in the 192 oscillator model yielded similar results: subnetwork (A) activity correlated negatively with increased FPC-DA connection strength ($r = -0.216$, $p = 0.001$), and subnetwork (B) activity correlated positively with increased FPC-DA connection strength($r = 0.538$, $p < 0.0001$). *(C)* These results could not be replicated in the 400 oscillator model, which accounts for all brain regions.

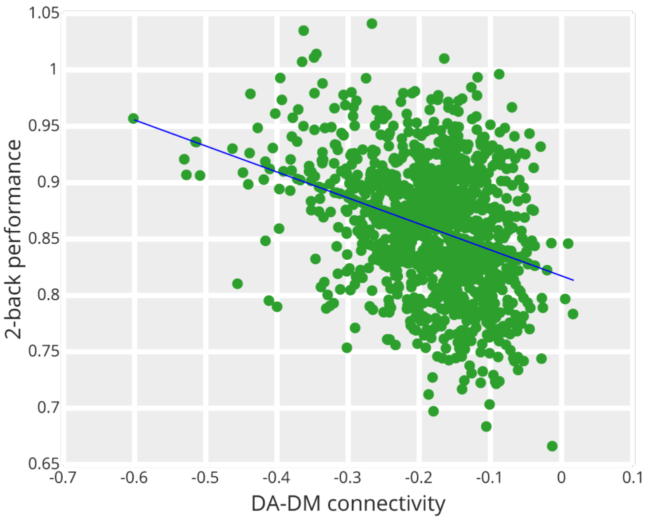

Supplementary Figure 15: **The strength of functional coupling between the dorsal attention and default mode systems relates to behavior.** The strength of the functional connection between the default mode and dorsal attention systems is anti-correlated with behavioral performance (Pearson's correlation coefficient $r = -0.8544$, $p < 0.0001$).

References

[1] Clare Kelly, A. M., Uddin, L. Q., Biswal, B. B., Castellanos, F. X. & Milham, M. P. Competition between functional brain networks mediates behavioral variability. *NeuroImage* **39**, 527–537 (2008).

[2] Delaveau, P. *et al.* Default mode and task-positive networks connectivity during the N-Back task in remitted depressed patients with or without emotional residual symptoms. *Human Brain Mapping* **3501**, 3491–3501 (2017).

[3] Boveroux, P., Vanhaudenhuyse, A. & Phillips, C. Breakdown of within- and between-network Resting State during Propofol-induced Loss of Consciousness. *Anesthesiology* **113**, 1038–1053 (2010).

[4] Hannawi, Y., Lindquist, M. A., Caffo, B. S., Sair, H. I. & Stevens, R. D. Resting brain activity in disorders of consciousness: a systematic review and meta-analysis. *Neurology* **84**, 1272–1280 (2015).

[5] Dixon, M. L., Andrews-Hanna, J. R., Spreng, R. N., Irving, Z. C. & Christoff, K. Anticorrelation between default and dorsal attention networks varies across default subsystems and cognitive states. *NeuroImage* **147**, 056424 (2017). URL `http://dx.doi.org/10.1016/j.neuroimage.2016.12.073` `http://biorxiv.org/content/early/2016/06/01/056424.abstract`.

[6] Xin, F. & Lei, X. Competition between frontoparietal control and default networks supports social working memory and empathy. *Social Cognitive and Affective Neuroscience* **10**, 1144–1152 (2014).

[7] Hearne, L., Cocchi, L., Zalesky, A. & Mattingley, J. B. Interactions between default mode and control networks as a function of increasing cognitive reasoning complexity. *Human Brain Mapping* **36**, 2719–2731 (2015).

[8] Spreng, R. N., Stevens, W. D., Viviano, J. D. & Schacter, D. L. Attenuated anticorrelation between the default and dorsal attention networks with aging: ev-

idence from task and rest. *Neurobiology of Aging* **45**, 149–160 (2016). URL http://dx.doi.org/10.1016/j.neurobiolaging.2016.05.020. 15334406.

[9] Hellyer, P. J. *et al.* The Control of Global Brain Dynamics: Opposing Actions of Frontoparietal Control and Default Mode Networks on Attention. *Journal of Neuroscience* **34**, 451–461 (2014). URL http://www.jneurosci.org/cgi/doi/10.1523/JNEUROSCI.1853-13.2014.

[10] Elton, A. & Gao, W. Divergent task-dependent functional connectivity of executive control and salience networks. *Cortex* **51**, 56–66 (2014). URL http://dx.doi.org/10.1016/j.cortex.2013.10.012.

[11] Christoff, K., Gordon, A. M., Smallwood, J., Smith, R. & Schooler, J. W. Experience sampling during fMRI reveals default network and executive system contributions to mind wandering. *Proceedings of the National Academy of Sciences* **106**, 8719–8724 (2009). URL http://www.pnas.org/cgi/doi/10.1073/pnas.0900234106. 0402594v3.

[12] Sridharan, D., Levitin, D. J. & Menon, V. A critical role for the right fronto-insular cortex in switching between central-executive and default-mode networks. *Proceedings of the National Academy of Sciences* **105**, 12569–12574 (2008). URL http://www.pnas.org/cgi/doi/10.1073/pnas.0800005105. arXiv:1408.1149.

[13] Popa, D., Popescu, A. T. & Pare, D. Contrasting Activity Profile of Two Distributed Cortical Networks as a Function of Attentional Demands. *Journal of Neuroscience* **29**, 1191–1201 (2009). URL http://www.jneurosci.org/cgi/doi/10.1523/JNEUROSCI.4867-08.2009.

[14] Spreng, R. N., Stevens, W. D., Chamberlain, J. P., Gilmore, A. W. & Schacter, D. L. Default network activity, coupled with the frontoparietal control network, supports goal-directed cognition. *NeuroImage* **53**, 303–317 (2010).

[15] Gao, W., Lin, W., Hill, C., Hill, C. & Carolina, N. Frontal Parietal Control Network Regulates the Anti-Correlated Default and Dorsal Attention Networks. *Human Brain Mapping* **33**, 192–202 (2012).

[16] Gerlach, K. D., Spreng, R. N., Gilmore, A. W. & Schacter, D. L. NeuroImage Solving future problems : Default network and executive activity associated

with goal-directed mental simulations. *NeuroImage* **55**, 1816–1824 (2011). URL http://dx.doi.org/10.1016/j.neuroimage.2011.01.030.

[17] Smallwood, J., Brown, K., Baird, B. & Schooler, J. W. Cooperation between the default mode network and the frontal-parietal network in the production of an internal train of thought. *Brain Research* **1428**, 60–70 (2012). URL http://dx.doi.org/10.1016/j.brainres.2011.03.072.

[18] Andrews-Hanna, J. R., Smallwood, J. & Spreng, R. N. The default network and self-generated thought: Component processes, dynamic control, and clinical relevance. *Annals of the New York Academy of Sciences* **1316**, 29–52 (2014). NIHMS150003.

[19] Bassett, D. S., Yang, M., Wymbs, N. F. & Grafton, S. T. Learning-induced autonomy of sensorimotor systems. *Nature neuroscience* **18**, 744–51 (2015). URL http://www.ncbi.nlm.nih.gov/pubmed/25849989.

[20] Lynall, M. E. *et al.* Functional connectivity and brain networks in schizophrenia. *J Neurosci* **30**, 9477–9487 (2010).

[21] Ceko, M. *et al.* Is a Responsive Default Mode Network Required for Successful Working Memory Task Performance? *Journal of Neuroscience* **35**, 11595–11605 (2015). URL http://www.jneurosci.org/cgi/doi/10.1523/JNEUROSCI.0264-15.2015.

[22] Mayer, J. S., Roebroeck, A., Maurer, K. & Linden, D. E. J. Specialization in the default mode: Task-induced brain deactivations dissociate between visual working memory and attention. *Human Brain Mapping* **31**, 126–139 (2010).

[23] Anticevic, A., Repovs, G., Shulman, G. L. & Barch, D. M. When less is more: TPJ and default network deactivation during encoding predicts working memory performance. *NeuroImage* **49**, 2638–2648 (2010). URL http://dx.doi.org/10.1016/j.neuroimage.2009.11.008.

[24] Anticevic, A. *et al.* The role of default network deactivation in cognition and disease. *Trends in Cognitive Sciences* **16**, 584–592 (2012). URL http://dx.doi.org/10.1016/j.tics.2012.10.008. NIHMS150003.

[25] Cole, M. W. *et al.* Multi-task connectivity reveals flexible hubs for adaptive task control. *Nature Neuroscience* **16**, 1348–1355 (2013).

[26] Fornito, A., Harrison, B. J., Zalesky, A. & Simons, J. S. Competitive and cooperative dynamics of large-scale brain functional networks supporting recollection. *Proceedings of the National Academy of Sciences* **109**, 12788–12793 (2012). URL http://www.pnas.org/cgi/doi/10.1073/pnas.1204185109. arXiv:1408.1149.

[27] Cocchi, L., Zalesky, A., Fornito, A. & Mattingley, J. B. Dynamic cooperation and competition between brain systems during cognitive control. *Trends in Cognitive Sciences* **17**, 493–501 (2013). URL http://dx.doi.org/10.1016/j.tics.2013.08.006.

[28] Snodgrass, J. G. & Corwin, J. Pragmatics of Measuring Recognition Memory: Applications to Dementia and Amnesia. *Journal of Experimental Psychology: General* **117**, 34–50 (1988).

[29] Satterthwaite, T. D. *et al.* Functional maturation of the executive system during adolescence. *The Journal of neuroscience : the official journal of the Society for Neuroscience* **33**, 16249–61 (2013). URL http://www.pubmedcentral.nih.gov/articlerender.fcgi?artid=3792462&tool=pmcentrez&rendert

[30] Eickhoff, S. B., Thirion, B., Varoquaux, G. & Bzdok, D. Connectivity-based parcellation: Critique and implications. *Human Brain Mapping* **36**, 4771–4792 (2015).

[31] Yeo, B. T. T. *et al.* The organization of the human cerebral cortex estimated by intrinsic functional connectivity. *J Neurophysiol* **106**, 1125–1165 (2011).

[32] Blumensath, T. *et al.* Spatially constrained hierarchical parcellation of the brain with resting-state fMRI. *NeuroImage* **76**, 313–324 (2013). URL http://dx.doi.org/10.1016/j.neuroimage.2013.03.024. NIHMS150003.

[33] Schaefer, A. *et al.* Local-Global Parcellation of the Human Cerebral Cortex from Intrinsic Functional Connectivity MRI. *Cerebral Cortex* **28**, 1–20 (2017). URL https://academic.oup.com/cercor/article-lookup/doi/10.1093/cercor/bhx179.

[34] Iacoboni, M. *et al.* Watching social interactions produces dorsomedial prefrontal and medial parietal BOLD fMRI signal increases compared to a resting baseline. *NeuroImage* **21**, 1167–1173 (2004). 1511.05762.

[35] Mars, R. B. *et al.* On the relationship between the "default mode network" and the "social brain". *Frontiers in Human Neuroscience* **6**, 1–9 (2012). URL http://journal.frontiersin.org/article/10.3389/fnhum.2012.00189/abstract.

[36] Kane, M. J. & Engle, R. W. The role of prefrontal cortex in working-memory capacity, executive attention, and general fluid intelligence: An individual-differences perspective. *Psychonomic Bulletin& Review* **9**, 637–671 (2002). arXiv:1011.1669v3.

[37] Curtis, C. E. & D'Esposito, M. Persistent activity in the prefrontal cortex during working memory. *Trends in Cognitive Sciences* **7**, 415–423 (2003).

[38] Balconi, M. Dorsolateral prefrontal cortex, working memory and episodic memory processes: Insight through transcranial magnetic stimulation techniques. *Neuroscience Bulletin* **29**, 381–389 (2013).

[39] Wang, H. E. *et al.* A systematic framework for functional connectivity measures. *Frontiers in Neuroscience* **8**, 1–22 (2014).

[40] Sun, F. T., Miller, L. M. & D'Esposito, M. Measuring interregional functional connectivity using coherence and partial coherence analyses of fMRI data. *NeuroImage* **21**, 647–58 (2004). URL http://www.ncbi.nlm.nih.gov/pubmed/14980567.

[41] Bassett, D. S. *et al.* Task-based core-periphery organization of human brain dynamics. *PLoS computational biology* **9**, e1003171 (2013). URL http://www.pubmedcentral.nih.gov/articlerender.fcgi?artid=3784512&tool=pmcentrez&rendert

[42] Murphy, A. C. *et al.* Explicitly Linking Regional Activation and Function Connectivity: Community Structure of Weighted Networks with Continuous Annotation. *arXiv* 1611.07962 (2016). URL http://arxiv.org/abs/1611.07962. 1611.07962.

[43] Pantazatos, S. P. & Li, X. Commentary: BRAIN NETWORKS. Correlated Gene Expression Supports Synchronous Activity in Brain Networks. Science 348, 1241–4. *Frontiers in neuroscience* **11**, 412 (2017).

[44] Eden, E., Navon, R., Steinfeld, I., Lipson, D. & Yakhini, Z. GOrilla: a tool for discovery and visualization of enriched GO terms in ranked gene lists. *BMC bioinformatics* **10**, 48 (2009).

[45] Supek, F., Bošnjak, M., Škunca, N. & Šmuc, T. REVIGO summarizes and visualizes long lists of gene ontology terms. *PloS one* **6**, e21800 (2011).

[46] Honey, C. J. *et al.* Predicting human resting-state functional connectivity from structural connectivity. *Proc Natl Acad Sci U S A* **106**, 2035–2040 (2009).

[47] Avena-Koenigsberger, A., Misic, B. & Sporns, O. Communication dynamics in complex brain networks (2018). URL `http://dx.doi.org/10.1038/nrn.2017.149`.

[48] Mason, A. Function in the human connectome. *NeuroImage* **19**, 389–399 (2009). `NIHMS150003`.

[49] Breakspear, M. Dynamic models of large-scale brain activity. *Nature neuroscience* **20**, 340–352 (2017). URL `http://www.ncbi.nlm.nih.gov/pubmed/28230845`.

[50] Messe, A., Rudrauf, D., Benali, H. & Marrelec, G. Relating structure and function in the human brain: relative contributions of anatomy, stationary dynamics, and non-stationarities. *PLoS Comput Biol* **10**, e1003530 (2014).

[51] Cabral, J., Kringelbach, M. L. & Deco, G. Functional connectivity dynamically evolves on multiple time-scales over a static structural connectome: Models and mechanisms. *NeuroImage* **160**, 84–96 (2017). URL `http://dx.doi.org/10.1016/j.neuroimage.2017.03.045`.

[52] Cabral, J., Kringelbach, M. L. & Deco, G. Exploring the network dynamics underlying brain activity during rest. *Progress in Neurobiology* **114**, 102–131 (2014). URL `http://dx.doi.org/10.1016/j.pneurobio.2013.12.005`.

[53] Deco, G., Jirsa, V. K. & McIntosh, A. R. Emerging concepts for the dynamical organization of resting-state activity in the brain. *Nature Reviews Neuroscience* **12**, 43–56 (2011). URL `http://dx.doi.org/10.1038/nrn2961`.

[54] Sporns, O. The human connectome: A complex network. *Annals of the New York Academy of Sciences* **1224**, 109–125 (2011).

[55] Muldoon, S. F. *et al.* Stimulation-Based Control of Dynamic Brain Networks. *PLoS Computational Biology* **12** (2016). `1601.00987`.

[56] Spiegler, A., Hansen, E. C., Bernard, C., McIntosh, A. R. & Jirsa, V. K. Selective activation of resting-state networks following focal stimulation in a connectome-based network model of the human brain. *eNeuro* **3** (2016).

[57] Gu, S. *et al.* Controllability of structural brain networks. *Nature Communications* **6**, 8414 (2015). URL `http://www.nature.com/doifinder/10.1038/ncomms9414`.

[58] Kuramoto, Y. *Chemical oscillations, waves, and turbulence* (Courier Corporation, 2003).

[59] Rodrigues, F. A., Peron, T. K. D. M., Ji, P. & Kurths, J. The Kuramoto model in complex networks. *Physics Reports* **610**, 1–98 (2016).

[60] Schmidt, R., LaFleur, K. J. R., de Reus, M. A., van den Berg, L. H. & van den Heuvel, M. P. Kuramoto model simulation of neural hubs and dynamic synchrony in the human cerebral connectome. *BMC neuroscience* **16**, 54 (2015).

[61] Senden, M., Reuter, N., van den Heuvel, M. P., Goebel, R. & Deco, G. Cortical rich club regions can organize state-dependent functional network formation by engaging in oscillatory behavior. *NeuroImage* **146**, 561–574 (2017). URL `http://dx.doi.org/10.1016/j.neuroimage.2016.10.044`. NIHMS150003.

[62] Cabral, J., Hugues, E., Sporns, O. & Deco, G. Role of local network oscillations in resting-state functional connectivity. *Neuroimage* **57**, 130–139 (2011).

[63] Ponce-Alvarez, A. *et al.* Resting-state temporal synchronization networks emerge from connectivity topology and heterogeneity. *PLoS computational biology* **11**, e1004100 (2015).

[64] Gollo, L. L., Roberts, J. A. & Cocchi, L. Mapping how local perturbations influence systems-level brain dynamics. *Neuroimage* **160**, 97–112 (2017).

[65] Cocchi, L. *et al.* A hierarchy of timescales explains distinct effects of local inhibition of primary visual cortex and frontal eye fields. *Elife* **5**, e15252 (2016).
