# Supplementary material for: Multimodal network dynamics underpinning working memory
Source: Nat Commun. 2020 Jun 15;11:3035. doi: 10.1038/s41467-020-15541-0 (PMC7295998; doi:10.1038/s41467-020-15541-0)
Supplement: Supplementary file 2 — Reporting Summary [file 41467_2020_15541_MOESM2_ESM.pdf]

nature research

Corresponding author(s): Danielle S. Bassett

Last updated by author(s): 1/30/2020

Reporting Summary

Nature Research wishes to improve the reproducibility of the work that we publish. This form provides structure for consistency and transparency in reporting. For further information on Nature Research policies, see Authors & Referees and the Editorial Policy Checklist .

Statistics

For all statistical analyses, confirm that the following items are present in the figure legend, table legend, main text, or Methods section.

n/a	Confirmed	
☐	☒	The exact sample size (*n*) for each experimental group/condition, given as a discrete number and unit of measurement
☐	☒	A statement on whether measurements were taken from distinct samples or whether the same sample was measured repeatedly
☐	☒	The statistical test(s) used AND whether they are one- or two-sided<br>*Only common tests should be described solely by name; describe more complex techniques in the Methods section.*
☐	☒	A description of all covariates tested
☐	☒	A description of any assumptions or corrections, such as tests of normality and adjustment for multiple comparisons
☐	☒	A full description of the statistical parameters including central tendency (e.g. means) or other basic estimates (e.g. regression coefficient) AND variation (e.g. standard deviation) or associated estimates of uncertainty (e.g. confidence intervals)
☐	☒	For null hypothesis testing, the test statistic (e.g. *F*, *t*, *r*) with confidence intervals, effect sizes, degrees of freedom and *P* value noted<br>*Give P values as exact values whenever suitable.*
☐	☒	For Bayesian analysis, information on the choice of priors and Markov chain Monte Carlo settings
☐	☒	For hierarchical and complex designs, identification of the appropriate level for tests and full reporting of outcomes
☐	☒	Estimates of effect sizes (e.g. Cohen's *d*, Pearson's *r*), indicating how they were calculated

Our web collection on statistics for biologists contains articles on many of the points above.

Software and code

Policy information about availability of computer code

Data collection	Data included in this study is part of the open source Human Connectome Project database.
Data analysis	All analysis code is custom written in Matlab and is freely available from the authors upon request.

For manuscripts utilizing custom algorithms or software that are central to the research but not yet described in published literature, software must be made available to editors/reviewers. We strongly encourage code deposition in a community repository (e.g. GitHub). See the Nature Research guidelines for submitting code & software for further information.

Data

Policy information about availability of data

All manuscripts must include a data availability statement. This statement should provide the following information, where applicable:

- Accession codes, unique identifiers, or web links for publicly available datasets
- A list of figures that have associated raw data
- A description of any restrictions on data availability

The datasets analyzed during the current study are available in the Human Connectome Project database: https://www.humanconnectome.org, as well as genetic expression data freely available from the Allen Brain Institute.

Field-specific reporting

Please select the one below that is the best fit for your research. If you are not sure, read the appropriate sections before making your selection.

☐ Life sciences ☒ Behavioural & social sciences ☐ Ecological, evolutionary & environmental sciences

Behavioural & social sciences study design

All studies must disclose on these points even when the disclosure is negative.

Study description	All analyses were quantitative measures of individual differences in human brain functional connectivity measured with fMRI, and individual differences in task performance on the n-back working memory task. Genetic data included genetic coexpression between a selection of genes across the brain.
Research sample	All magnetic resonance imaging data analyzed is included in the Human Connectome Project S900 Release, all genetic expression data is available from the Allen Brain Institute
Sampling strategy	No sample size calculation was employed.
Data collection	All data was independently collected by the Human Connectome Project or the Allen Brain Institute. We analyzed all subjects that were part of the S900 release, as well as 6 postmortem samples
Timing	Data was collected between 2009 and 2015.
Data exclusions	We excluded all subjects in the S900 release that did not have all 4 of the following data: working memory task, resting state functional magnetic resonance scan, high resolution anatomical scan, diffusion tensor imaging scan. After excluding these subjects, our sample included 644 subjects.
Non-participation	For the fMRI data analyzed, only subjects that underwent (1) the working memory task, (2) resting state functional imaging, (3) high resolution anatomical imaging, and (4) diffusion tensor imaging were included. After excluding all subjects that did not complete each of these components, we were left with 644 subjects.
Randomization	Subjects were not randomized to different groups.

Reporting for specific materials, systems and methods

We require information from authors about some types of materials, experimental systems and methods used in many studies. Here, indicate whether each material, system or method listed is relevant to your study. If you are not sure if a list item applies to your research, read the appropriate section before selecting a response.

Materials & experimental systems

n/a	Involved in the study	
☒	☐	Antibodies
☒	☐	Eukaryotic cell lines
☒	☐	Palaeontology
☒	☐	Animals and other organisms
☐	☒	Human research participants
☒	☐	Clinical data

Methods

n/a	Involved in the study	
☒	☐	ChIP-seq
☒	☐	Flow cytometry
☐	☒	MRI-based neuroimaging

Human research participants

Policy information about studies involving human research participants

Population characteristics	See above for details. Furthermore, extensive details on population characteristics for the sample of this study can be found here: https://www.ncbi.nlm.nih.gov/pmc/articles/PMC3724347/
Recruitment	All subjects were recruited independently by the Human Connectome project. Details can be found here: https://www.ncbi.nlm.nih.gov/pmc/articles/PMC3724347/
Ethics oversight	All analyses were performed in accordance with the relevant ethical regulations of the WU-Minn HCP Consortium Open Access Data Use Terms. Informed consent was obtained in writing from all participants.

Note that full information on the approval of the study protocol must also be provided in the manuscript.

Magnetic resonance imaging

Experimental design

Design type	All analyses were executed on the preprocessed time series. No event related designs or block designs were used.

Design specifications	The following descriptions for each task have been adapted for brevity from the Human Connectome Project Manual. Working Memory. The category specific representation task and the working memory task are combined into a single task paradigm. Participants were presented with blocks of trials that consisted of pictures of places, tools, faces and body parts (non-mutilated parts of bodies with no "nudity"). Within each run, the 4 different stimulus types were presented in separate blocks. Also, within each run, 1⁄2 of the blocks use a 2-back working memory task and 1⁄2 use a 0-back working memory task (as a working memory comparison). A 2.5 second cue indicates the task type (and target for 0-back) at the start of the block. Each of the two runs contains 8 task blocks (10 trials of 2.5 seconds each, for 25 seconds) and 4 fixation blocks (15 seconds). On each trial, the stimulus is presented for 2 seconds, followed by a 500 ms inter-task interval (ITI).
Behavioral performance measures	All performance measures were chosen a priori. In the working memory tasks, we used the mean accuracy across all n-back conditions (face, body, place, tool), as well as the d-prime statistic.

Acquisition

Imaging type(s)	Functional
Field strength	3T
Sequence & imaging parameters	The acquisition parameters for each data type are as follows. The parameters for the acquisition of high-resolution structural scan were: TR = 2400 ms, TE = 2.14 ms, TI = 1000 ms, flip angle = 8 deg, FOV = 224 × 224 mm, voxel size = 0.7 mm isotropic, BW = 210 Hz/Px, acquisition time = 7:40 minutes. Functional magnetic resonance images were collected during both rest and task with the following parameters: TR = 720 ms, TE = 33.1 ms, flip angle = 52 deg, FOV = 208×180 mm, matrix = 104×90, slice thickness = 2.0 mm, number of slices = 72 (2.0 mm isotropic), multi factor band = 8, echo spacing = 0.58 ms.
Area of acquisition	Whole brain

Diffusion MRI ☒ Used ☐ Not used

Parameters	Diffusion tensor images were collected with the following parameters: TR = 5520 ms, TE = 89.5 ms, flip angle = 78 deg, refocusing flip angle = 160 deg, FOV = 210×180, matrix = 168×144, slice thickness = 1.25 mm, number of slices = 111 (1.25 mm isotropic), multiband factor = 3, echo spacing = 0.78 ms, b-vaues = 1000, 2000, and 3000 s/mm2.

Preprocessing

Preprocessing software	Analysis of Functional NeuroImages (AFNI), FSL. Preprocessing steps are extensively documented here: https://www.sciencedirect.com/science/article/pii/S1053811913005053
Normalization	Registration of the T1 to atlas space includes an initial volumetric registration to MNI152 space using FSL's linear FLIRT tool, followed by the nonlinear FNIRT algorithm.
Normalization template	MNI152
Noise and artifact removal	For both resting-state and task functional connectivity, CompCor, with five principal components from the ventricles and white matter masks, was used to regress out nuisance signals from the time series. In addition, the 12 detrended motion estimates provided by the Human Connectome Project were regressed out from the time series. The mean global signal was removed and then time series were band-pass filtered from 0.009 to 0.08 Hz.
Volume censoring	Frames with greater than 0.2 mm frame-wise displacement or a derivative root mean square (DVARS) above 75 were removed as outliers. Sessions composed of greater than 50 percent outlier frames were not further analyzed.

Statistical modeling & inference

Model type and settings	Functional connectivity
Effect(s) tested	Pearson r correlations between all ROIs

Specify type of analysis: ☐ Whole brain ☒ ROI-based ☐ Both

Anatomical location(s)	We parcellated the brain into 400 discrete and non-overlapping regions of interest using the Schaefer atlas (fslr32k surface) [69]. Notably, the Schaefer atlas was originally validated in the same HCP data that we study here, and it yields a functional demarcation of both the default mode and the frontoparietal systems. Of course other functionally defined atlases exist, but they are less ideal for our purposes for several reasons; the Power atlas [64] does not provide full cortical coverage, and the Gordon [40] and Brainnetome [31] atlases are lower spatial resolution including 333 and 246 regions, respectively. The Schaefer atlas provides an assignment of each region to one of 17 putative cognitive systems: two visual, two somatomotor, two dorsal attention, two salience/ventral attention, one limbic, three frontoparietal, three default mode, and one temporo-parietal system. To ensure that the granularity of the data was consistent with the granularity of our hypotheses, we collapsed these 17 systems into 8 systems by combining individual systems that belonged to the same cognitive system; that is, we combined the two visual systems into a single system, the two somatomotor systems into a single system, the two dorsal attention systems into a single system, the two salience systems into a single system, the three

frontoparietal systems into a single frontoparietal system, and the three default mode systems into a single system.

Statistic type for inference (See Eklund et al. 2016)	Pearson r correlations were calculated between all ROIs
Correction	No multiple comparisons were calculated for the Pearson correlations.

Models & analysis

n/a	Involved in the study
☐	☒ Functional and/or effective connectivity
☐	☒ Graph analysis
☐	☒ Multivariate modeling or predictive analysis

Functional and/or effective connectivity

Pearson r correlation

Graph analysis

Subject level weighted graphs were used. Weighted node degree was calculated for each subject.

Multivariate modeling and predictive analysis

At several points, we calculate the statistical difference between outcome variables of the two sub-networks. To that end, we initially take a parametric approach, and then we confirm all of our findings using a non-parametric permutation-based approach. In all visualizations of statistical relationships, subject effects have been regressed out from the dependent variable.

In testing our hypotheses, we often asked questions of the following form: Does the strength of the connection between subnetwork (A) and the default mode system differ from the strength of the connection between subnetwork (B) and the default mode system? For questions of this form, we used a multilevel model where each outcome variable (e.g., a measurement of connection strength) has attributes encoding subnetwork membership, task run, and subject identity. The multilevel model framework [74] accounts for the nested nature of the data (multiple scans nested within subject). We specified the model as: OutcomeVariableit = B0i + B1iSubNetworkit + eit,
where OutcomeVariableit is the outcome variable (i.e. connection strength) for person i on run t; B0i indicates the level of the outcome in subnetwork (A); B1i indicates differences in the level of outcome associated with subnetwork (B) versus subnetwork (A); and eit are residuals.
Person-specific intercepts (from Level 1) were specified (at Level 2) as: B0i = γ00 + u0i, and B1i = γ10, where γ denotes a sample-level parameter and u denotes residual between-person differences that may be correlated, but are uncorrelated with eit. The multilevel model was fit with lme in R using maximum likelihood estimation. In the case of many outliers, we treat our data with robust models, rather than standard linear models. Robust models down-weight points of data, where the most outlying points are down-weighted most severely. Specifically, we implement robust multilevel models using robustlmm in R [48]. We note in the text whenever a robust multilevel model is used.

Unless otherwise noted, we use a repeated measures correlation when examining the association be-tween two continuous variables [7]. The repeated measures correlation accounts for non-independence among observations (due to multiple runs per subject) by using a form of analysis of covariance (AN-COVA) to adjust for between-person variance. The model is specified as: Measure1it = Measure2i + Subjecti + c(barMeasure2i) + eit, where barMeasure1it is the value of variable one for subject i during measurement occasion t, barMeasure2i is the mean value of the second variable in the i-th participant, Subjecti is a unique identifier for each participant, and c(Measure2i) is the covariate for the i-th participant and is equal to B(Measure2it − Measure2i), where B is the slope coefficient of the covariate. Like a Pearson correlation coefficient (r), the repeated measures correlation (rrm) is bounded by -1 to 1, and represents the strength of the linear association between two variables. The repeated measures correlation was estimated using the rmcorr package in R [7].

In addition to the multilevel linear model, we employ a complementary permutation-based approach. We begin with vectors Y1 ∈ R1×4n, Y2 ∈ R1×4n, and S ∈ R1×4n, and we wish to test whether there is a difference in the means of Y1 and Y2 against a null model. To construct the null model, for subject i we find the two entries J = (j1,j2) for which S = i. We then randomly reassign elements J between Y and Y , and repeat this procedure for all subjects to construct a null Y null 121 and null Y null where we would expect the means to be equal. We then calculate the mean difference 2 d = mean(Y null − Y null). We re-permute and recalculate the mean 10000 times to establish a null 1 2 null distribution of the difference, and we determine a p-value for the true effect by calculating the proportion of null differences that are greater than the observed difference.
